# The wheat WRKY transcription factors TaWRKY49 and TaWRKY62 confer differential high-temperature seedling-plant resistance to *Puccinia striiformis* f. sp. *tritici*

**DOI:** 10.1371/journal.pone.0181963

**Published:** 2017-07-25

**Authors:** Junjuan Wang, Fei Tao, Wei Tian, Zhongfeng Guo, Xianming Chen, Xiangming Xu, Hongsheng Shang, Xiaoping Hu

**Affiliations:** 1 State Key Laboratory of Crop Stress Biology for Arid Areas, College of Plant Protection, Northwest A&F University, Yangling, Shaanxi, China; 2 Agricultural Research Service, Department of Agriculture and Department of Plant Pathology, Washington State University, Pullman, Washington, United States of America; 3 NIAB East Malling Research, East Malling, Kent, United Kingdom; GERMANY

## Abstract

WRKY transcription factors (TFs) play crucial roles in plant resistance responses to pathogens. Wheat stripe rust, caused by the fungal pathogen *Puccinia striiformis* f. sp. *tritici* (*Pst*), is a destructive disease of wheat (*Triticum aestivum*) worldwide. In this study, the two WRKY genes *TaWRKY49* and *TaWRKY62* were originally identified in association with high-temperature seedling-plant resistance to *Pst* (HTSP) resistance in wheat cultivar Xiaoyan 6 by RNA-seq. Interestingly, the expression levels of *TaWRKY49* and *TaWRKY62* were down- and up-regulated, respectively, during HTSP resistance in response to *Pst*. Silencing of *TaWRKY49* enhanced whereas silencing *TaWRKY62* reduced HTSP resistance. The enhanced resistance observed on leaves following the silencing of *TaWRKY49* was coupled with increased expression of salicylic acid (SA)- and jasmonic acid (JA)-responsive genes *TaPR1*.*1* and *TaAOS*, as well as reactive oxygen species (ROS)-associated genes *TaCAT* and *TaPOD*; whereas the ethylene (ET)-responsive gene *TaPIE1* was suppressed. The decreased resistance observed on leaves following *TaWRKY62* silencing was associated with increased expression of *TaPR1*.*1* and *TaPOD*, and suppression of *TaAOS* and *TaPIE1*. Furthermore, SA, ET, MeJA (methyl jasmonate), hydrogen peroxide (H_2_O_2_) and abscisic acid (ABA) treatments increased *TaWRKY62* expression. On the other hand, MeJA did not affect the expression of *TaWRKY49*, and H_2_O_2_ reduced *TaWRKY49* expression. In conclusion, *TaWRKY49* negatively regulates while *TaWRKY62* positively regulates wheat HTSP resistance to *Pst* by differential regulation of SA-, JA-, ET and ROS-mediated signaling.

## Introduction

Plants initiate pathogen-associated molecular pattern (PAMP)-triggered immunity (PTI) responses to recognize molecular signatures of many pathogens via pattern recognition receptors (PRRs). Pathogens deliver virulence effectors to suppress plant PTI while the plant detects these effectors by resistance (R) proteins, activating effector-triggered immunity (ETI) responses [1]. R gene-mediated ETI involves complex defense processes, including production of reactive oxygen species (ROS) and salicylic acid (SA), rapid programmed cell death (hypersensitive responses, HR) and induction of host genes including pathogenesis-related (*PR*) genes [1]. These complex defense responses involve timely recognition of the invading pathogen, followed by defense responses through complicated signaling pathways [2]. These signaling pathways regulate the defense responses by fine-turning transcriptional activation of defense-related genes [3]. Increasing evidence has revealed that transcriptional regulation of gene expression in response to pathogen attacks is a crucial part of the plant defense system [3, 4]. Defense-related gene expression is regulated by transcriptional factors (TFs) that alter gene expression by binding to target DNA-binding sites of genes, in cooperation with other proteins. WRKY proteins are zinc-finger-containing TFs that belong to a large family of related proteins in the plant kingdom [5]. There are 109, 72 and more than 160 WRKY family members identified in rice [6], *Arabidopsis* [7] and wheat [8, 9], respectively. WRKY TFs are structurally classified into three main groups (I, II and III), and also multiple subgroups (e.g. IIa, IIb and IIc, etc.) that is dependent on the presence of one or two WRKY domains in a 60-amino acid region at the N-terminus, having the conserved heptapeptide sequence WRKYGQK, and a zinc-finger like motif at the C-terminus [5].

WRKY TFs have received increasing attention for their roles in regulating plant defense responses [6], including cell death [10]. The oxidative burst and production of reactive oxygen species (ROS), are among the earliest defense reactions that are activated in response to pathogen attack [11, 12], which may lead to rapid programmed cell death preventing pathogen progression [13]. When *Nicotiana tabacum NtWRKY1* is co-expressed with the salicylic acid-induced protein kinase (SIPK) there was significantly more host cell death than that observed with expression of SIPK alone in response to *Ralstonia solanacearum* [10]. Salicylic acid (SA), ethylene (ET) and jasmonic acid (JA) are the main signaling molecules involved in plant defense responses. In *Arabidopsis*, *AtWRKY70* is linked to SA-mediated signaling in response to *Erysiphe cichoracearum*, a biotrophic fungus, and JA-mediated signaling against *Alternaria brassicicola*, a necrotrophic fungus [14]. Recently, we demonstrated that *TaWRKY70* positively regulates high-temperature seedling-plant (HTSP) resistance to *Puccinia striiformis* f. sp. *tritici* (*Pst*) in wheat, probably through the SA- and ET-mediated signaling pathways [15]. WRKY TFs may also act as negative regulators of plant defense responses. Overexpressing *OsWRKY62* in rice compromises the basal defense and *Xa21* (receptor-like kinases)-mediated resistance against *Xanthomonas oryzae* pv. *oryzae* (*Xoo*), and suppresses the activation of defense-related genes [16].

Wheat stripe rust (or yellow rust), caused by *Pst*, is one of the most destructive wheat (*Triticum aestivum*) diseases worldwide [17, 18]. Temperature change-induced wheat resistance to *Pst* is usually non-race-specific and durable, of which two types of resistance were reported: high-temperature adult-plant (HTAP) and high-temperature seedling-plant (HTSP) resistance. Wheat plants with only HTAP resistance are susceptible to *Pst* in the seedling stage and under low temperature, but become resistant as plants grow old and under high temperature [19, 20]. In HTSP resistance, wheat seedlings are susceptible to *Pst* at low temperatures but become resistant when exposed to high temperatures for 24 h at the initial *Pst* symptom-expression stage (eight days after inoculation) [21–24]. There is little information whether WRKY TFs play a role in the HTSP resistance against *Pst* [15].

Recently, we obtained a cDNA library with RNA from wheat cultivar Xiaoyan 6 (possessing HTSP resistance) infected by the *Pst* CYR32 pre-exposure to a high temperature (HT) treatment [15°C for first eight days post-inoculation, then 20°C for 24 h, and then back to 15°C] and sequenced the library. Preliminary studies identified 24 differentially regulated candidate WRKY TFs during the HTSP process, including *TaWRKY70* [15]. In this study, we characterized two WRKY TFs in relation to their roles in the HTSP resistance to *Pst*: a WRKY62 homolog (the second most highly up-regulated TF, after *TaWRKY70*) named *TaWRKY62*, and a WRKY49 homolog (the only down-regulated TF), named *TaWRKY49*. We hypothesize that *TaWRKY62* and *TaWRKY49* play positive and negative roles in regulating the HTSP resistance, respectively. Functional analyses of these two WRKY genes by gene silencing experiments were performed, and further data were collected on the regulation of the genes when exposed to phytohormones and abiotic stressors.

## Materials and methods

### Plant materials, growth conditions, high-temperature resistance induction and stress treatments

Wheat cultivar Xiaoyan 6 and *Pst* race CYR32 were used to study the wheat-*Pst* interaction. The seeds of Xiaoyan 6 were provided by Dr. Sanhong Fan in Northwest A&F University, and the *Pst* CYR32 urediniospores were provided by Prof. Qiuzhen Jia in Institute of Plant Protection, Gansu Academy of Agricultural Science. Wheat seeds (10–15) were grown in a plastic pot (10×10×10 cm^3^) filled with a potting mixture under rust-free conditions. The first leaves of seedlings at the two-leaf stage (approximately 10–14 days after planting) were uniformly brushed with a mixture of *Pst* urediniospores and sterile water at a ratio of approximately 1:10–15 (v/v). The seedlings were then placed in a dew chamber in the dark for 24 h (temperature, 10°C; rh, 90–100%) and subsequently transferred to a growth chamber (Percival E-30B, Perry, IA, USA) and grown under 16 h of light at 15±1°C (rh, 60–80%; supplemented with sodium lighting (505 μmol/m^2^/s photon flux density)) and 8 h of dark at 12±1°C (rh, 60–80%). In parallel, control plants were brushed with sterile water. In the initial symptom-expression stage of rust development (8 dpi), the plants were divided into two groups for exposure to different temperature regimes. The first group was subjected to low-temperature (LT) treatment; i.e., *Pst*-inoculated wheat plants were incubated at a constant temperature (15±1°C). The second group was subjected to high temperature (HT) treatment, i.e., *Pst*-inoculated wheat plants were incubated at 15±1°C, at 8 days post inoculation transferred to a growth chamber set at 20±1°C and incubated for 24 h and then moved back to and maintained thereafter at 15±1°C, as the exposure to 20±1°C for 24 h was previously shown to activate HTSP resistance to *Pst* [23]. The leaf tissues from LT and HT treatments were sampled at 0, 48, 96, 192, 194, 198, 204, 216, 240, 264 and 312 hours post inoculation (hpi); the 192-hpi time corresponded to the beginning of HT treatment. Leaves, stems and root tissues were sampled also from the two-leaf-stage seedlings. Three biological replicates were used for each assay.

In addition, experiments were conducted to study wheat responses to extreme temperatures and to hormone treatments. For extreme-temperature treatments, two-leaf stage seedlings were incubated under cold (4°C) or hot (40°C) temperatures. For hormone treatments, seedlings were sprayed at 15°C with hydrogen peroxide (H_2_O_2_, 100 μM), methyl jasmonate (MeJA, 100 μM), ethylene (ET, 100 μM), salicylic acid (SA, 100 μM) or abscisic acid (ABA, 100 μM) [25]. In all of these treatments, seedlings in a similar state of growth (two-leaf stage) were used, and non-treated wheat seedlings were used as controls. All of the treated and non-treated seedlings were harvested at 0, 0.5, 2, 6, 12 and 24 h, frozen in liquid nitrogen and stored at –80°C. Three biological replicates were performed independently for each time point.

### Cloning and sequence analysis of *TaWRKY49* and *TaWRKY62*

Total RNA from leaf tissues was extracted using the PureLink^®^ Plant RNA Reagent (Invitrogen, Carlsbad, CA, USA). After genomic DNA contamination was removed through DNase I treatment (Thermo Fisher Scientific, Waltham, MA, USA), 500 ng of poly(A)+ mRNA was converted into cDNA using RevertAid M-MuLV reverse transcriptase (Thermo Fisher Scientific, Waltham, MA, USA). Based on RNA-seq data, a set of RACE primers GSP5 and GSP3 (S1 Table) targeting the 5' and 3’ ends were designed by Primer 5.0 software to amplify the complete cDNA of *TaWRKY49*. The 5' RACE was performed using the SMART RACE cDNA Amplification Kit (Clontech, Mountain View, CA, USA), and 3’ RACE using the 3’-Full RACE Core Set with PrimeScript^™^ RTase (TaKaRa, Tokyo, Japan). The complete cDNA of *TaWRKY62* was obtained by reverse transcription-PCR (RT-PCR) (primers TaWRKY62cDNA, S1 Table) based on the sequence from RNA-seq using 2×EasyTaq PCR SuperMix (+dye) (Transgen Biotech, Beijing, China). PCR products were extracted, combined with the pMD-18T plasmid (TaKaRa, Tokyo, Japan), and then sequenced. Amino acid and molecular weight predictions were conducted by EMBOSS (http://emboss.open-bio.org/wiki/Appdocs) and ExPASy (http://web.expasy.org/protparam/), respectively, subcellular localization was performed by Euk-mPLoc 2.0 (http://www.csbio.sjtu.edu.cn/bioinf/euk-multi-2/), gene mapping was analyzed via EnsemblPlants (http://plants.ensembl.org/Triticum_aestivum/Tools/Blast?db=core), alignment of amino acid sequence was performed by BLAST (http://www.ncbi.nlm.gov/blast) and DNAman software 5.2.2, and phylogenetic tree was constructed using MEGA4.0, and was performed as described by Wang et al. [15].

### Plasmid construction

The γRNA-based vector derived from BSMV was constructed as described previously [26]. cDNA fragments derived from the coding sequence (251 bp, nt 9–259) of *TaWRKY49* complete cDNA were used to construct the recombinant TaWRKY49-as plasmids, and cDNA fragments (149 bp, 593–741) derived from *TaWRKY62* were used to construct the recombinant TaWRKY62-as plasmids. All primers used for vector construction are listed in S1 Table.

### BSMV-mediated *TaWRKY49* and *TaWRKY62* silencing

Plasmids used for barley stripe mosaic virus (BSMV)-mediated gene silencing were constructed according to Holzberg et al. [26]. The cDNA fragments of *TaWRKY49* (251 bp) and *TaWRKY62* (149 bp) were amplified with primer pairs TaWRKY49VIGS and TaWRKY62VIGS respectively (S1 Table). The wheat PDS gene (*TaPDS*) was replaced with the cDNA fragment of *TaWRKY49* or *TaWRKY62* in BSMV:γ-PDS.

The inoculation and incubation conditions for the virus were as described previously by Scofield et al. [27] and Wang et al. [15] respectively. Rust symptoms and sporulation on the fourth leaves were assessed on 14 days post inoculation (dpi).

### Reverse Transcription Quantitative PCR (RT-qPCR)

Total RNA from wheat tissue was extracted using the PureLink^®^ Plant RNA Reagent (Invitrogen, Carlsbad, CA, USA), then genomic DNA contamination was removed using DNase I treatment (Thermo Fisher Scientific, Waltham, MA, USA), and 500 ng of poly(A)+ mRNA was reversed transcribed into cDNA using a PrimeScript^®^ RT Reagent Kit (TaKaRa, Tokyo, Japan). RT-qPCR was conducted to quantify the expression of *TaWRKY49* (primer TaWRKY49Q, S1 Table) and *TaWRKY62* (primer TaWRKY62Q, S1 Table) according to Wang et al. [15]. After a preliminary study (data not shown), wheat *26S* gene (ATP-dependent 26S proteasome regulatory subunit) (Unigene No. Ta22845) (primer Ta26SQ, S1 Table) was used as the internal reference for each RT-qPCR assay. The efficiency and specificity of the primer pairs are presented in S1 Fig, and the primers are given in S1 Table. The relative expression of mRNA was calculated using the 2^–ΔΔCt^ method [28].

### Silencing efficiency and expression levels of *TaWRKY49* and *TaWRKY62* under high-temperature treatment after *Pst* infection

The fourth leaves of the seedlings for which the second leaves had been pre-inoculated with BSMV:00 and BSMV:WRKY49-as or BSMV:WRKY62-as were sampled separately at 0, 24, 48 and 120 hours post inoculation (hpi) with CYR32. RT-qPCR was performed to determine the silencing efficiency of *TaWRKY49* and *TaWRKY62* in each assay.

To assess whether the expression level of *TaWRKY49* and *TaWRKY62* on their corresponding silenced plants with *Pst* infection could be induced by high temperature (HT) treatment, plants from the low temperature (LT) treatment were divided into two groups. One group was immediately subjected to the HT treatment at 192 hpi of *Pst*, and another group was kept under the LT treatment. Each group included *TaWRKY49* or *TaWRKY62*-silenced and BSMV:00 control plants. Samples were harvested at 0, 12, 24, 48, 72 and 120 h post-temperature treatment (hptt) from both LT and HT treatments to detect the temperature-induced expression of *TaWRKY49* and *TaWRKY62* via RT-qPCR. Stripe rust symptoms and *Pst* sporulation on leaves were assessed at 14 dpi. Additionally, the transcript levels of *TaPR1*.*1* (Genbank Accession AJ007348) (primer TaPR1.1Q, S1 Table), *TaAOS* (AY196004) (primer TaAOSQ, S1 Table), *TaPIE1* (EF583940) (primer TaPIE1Q, S1 Table), *TaCAT* (X94352) (primer TaCATQ, S1 Table) and *TaPOD* (TC303653) (primer TaPODQ, S1 Table) on BSMV:00 and *TaWRKY49* or *TaWRKY62* silenced leaves subjected to the LT and HT treatments were quantified and compared at 0, 12, 24, 48, 72 and 120 hptt through RT-qPCR. Three biological replicates were performed for each time point.

### Histological observations of *TaWRKY49*-silenced and *TaWRKY62*-silenced wheat plants under LT and HT treatments

Wheat leaves pre-infected with BSMV and subjected to the LT treatment after *Pst* inoculation were sampled at 48 and 120 hpi to observe *Pst* development and the host response on *TaWRKY49-*silenced and *TaWRKY62*-silenced plants. At eight days following *Pst* inoculation, BSMV-pre-infected wheat leaves subjected to the LT and HT treatments were sampled at 0 (192 hpi), 24, 48, 72 and 120 h to examine changes in *Pst* development and host response, arising from the temperature-reduced expression of *TaWRKY49* and temperature-induced expression of *TaWRKY62*. The staining and fixing of specimens were performed using Calcofluor M2R White New (Sigma, MO, US) staining method as described previously by Wang et al. [29]. After fading and fixation, cleared wheat leaf segments were analysed to determine the hyphal length and number of haustorial mother cells under a microscope. Autofluorescence of pathogen-induced host necrotic cells was observed under a fluorescence microscope (excitation filter, 485 nm; dichromic mirror, 510 nm; barrier filter, 520 nm). Given the lateness of initial *Pst* development, linear lengths of the fungal colonies and uredinia were chosen for the assessment of fungal development. Colony length was measured from the substomatal vesicle to the apex of the longest hypha, and uredinium length as the length of its long shaft. In addition, plant cell death was defined as the presence of autofluorescence under a fluorescence microscope associated with an infection unit. About 30–50 infection sites on 8–10 wheat leaf segments (length, 1.5 cm) from 8–10 randomly selected wheat plants were examined. All of the microscopic observations were performed using an Olympus BX-51 microscope (Olympus Corporation, Tokyo, Japan), and the data were analysed using DP-BSW software.

### Detection of H_2_O_2_ and O_2_^−^ (ROS)

The production of H_2_O_2_ in the *TaWRKY49*-silenced and *TaWRKY62*-silenced leaves after *Pst* infection at the treatment of HT or LT was analyzed histochemically using a 3,3-diaminobenzidine (DAB; Amresco, Solon, OH, USA) staining method [30]. The detection of O_2_^−^ was based on the nitroblue tetrazolium (NBT, Amresco, Solon, OH, USA) staining method [29, 31]. Leaf samples were harvested at the same time as those for the histopathological analysis. Before microscopic examinations, 6–8 leaf segments (1.5 cm) were randomly selected, fixed and decolorized as described above. The occurrence of brownish and blue colors in leaf tissue indicated the presence of H_2_O_2_ and O_2_^−^, respectively. The percentage of DAB or NBT staining is based on 30 infection sites that were randomly selected from one leaf segment, and the mean value of percentages of DAB or NBT staining come from 3–8 leaf segments.

### Statistical analyses

Analysis of variance was conducted using SAS v8.01 (SAS Institute Inc., Cary, NC, USA). Data relating to the number of necrotic cells and haustorial mother cells as well as length of hyphae in silenced and non-silenced wheat leaves under LT treatment were performed according to the Student’s *t*-test at *P* = 0.05 or *P* = 0.01, under the assumption of homogeneous variance. Data on hormone treatments as well as cold and heat stresses were analyzed using the Duncan’s multiple range test for different time points. Data relating to with or without *Pst* inoculation under LT or HT treatments, percentage of infection sites with DAB and NBT staining, number of necrotic plant cells, length of fungal colonies and uredinia for each time point were analyzed using the Duncan’s multiple range test. The relative expression of *TaWRKY49*, *TaPR1*.*1*, *TaAOS*, *TaPIE1*, *TaCAT* and *TaPOD* in the four VIGS treatments (HT BSMV:00, LT BSMV:00, HT BSMV:TaWRKY49-as and LT BSMV:TaWRKY49-as) as well as the relative expression of *TaWRKY62* and the *TaPR1*.*1*, *TaAOS*, *TaPIE1*, *TaCAT* and *TaPOD* genes in the four VIGS treatments were analyzed using Duncan’s multiple range test for each time point.

## Results

### Sequence analysis of *TaWRKY49* and *TaWRKY62*

**Fig 1:**
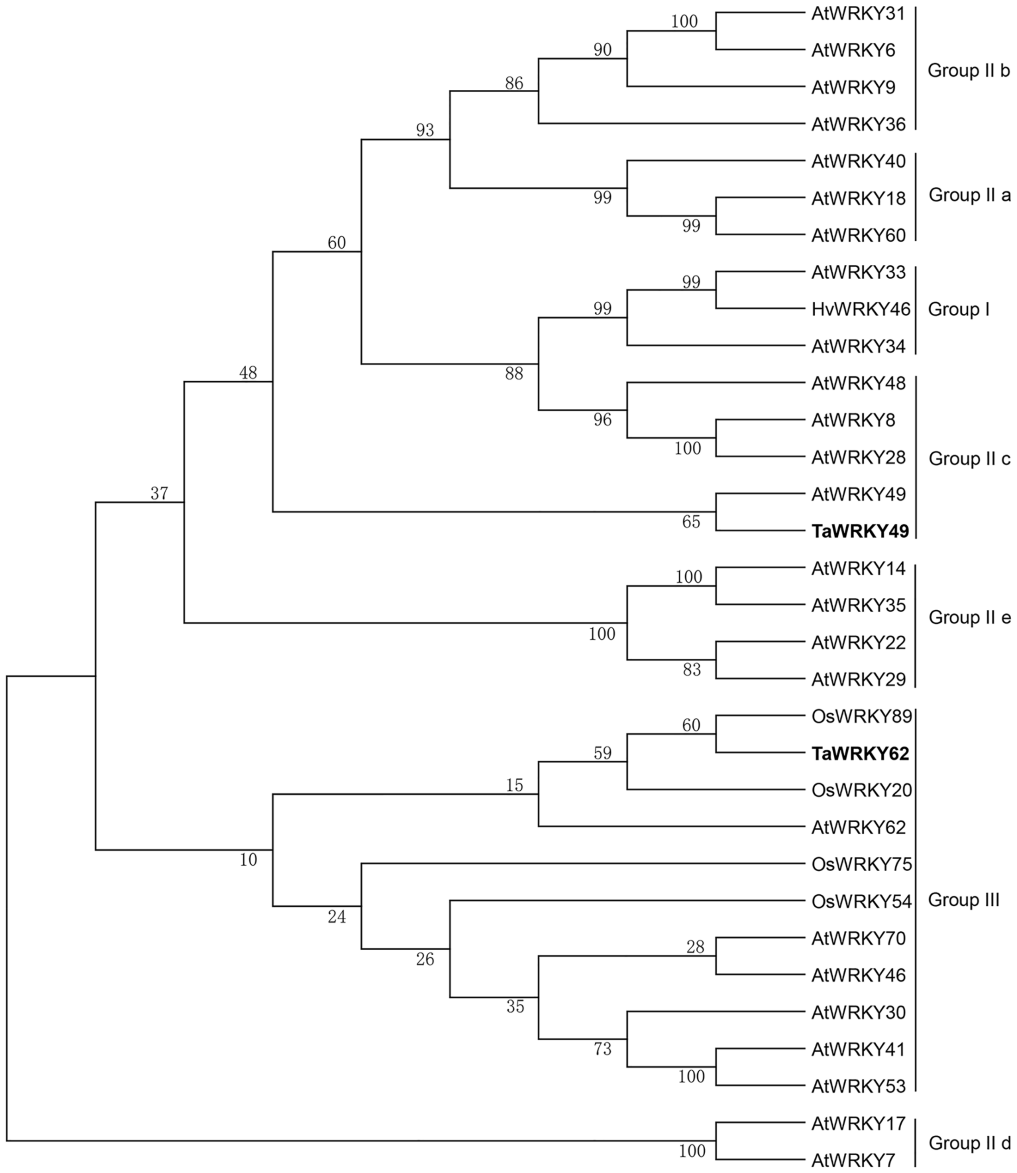
Dendrogram showing relationships of wheat TaWRKY49 and TaWRKY62 with other plant WRKY proteins. The GenBank accession numbers of the WRKY proteins used for constructing the phylogenetic tree are given in S2 Table. Ta, *Triticum aestivum*; At, *Arabidopsis thaliana*; Hv, *Hordeum vulgare*, Os, *Oryza sativa*. The phylogenetic tree was constructed with MEGA4.0 using a bootstrap test of phylogeny with a minimum evolution test and a parameter of 1000 replications.

Twenty-four WRKY TFs with differential expression during the HT treatment post *Pst* infection were identified from the preliminary RNA-seq data analysis. Among these TFs, *TaWRKY49* was the only one down-regulated (approximately 2.9 times), and *TaWRKY62* was the second most highly up-regulated (rank only second to *TaWRKY70* [15] (approximately 5.2 times). Two full-length 1221-bp cDNA and 798-bp cDNA were obtained respectively, from *Pst*-infected leaves. The cDNA of 1221-bp length encodes a 321-amino-acid polypeptide protein with a molecular mass of 34.17 kDa and a theoretical isoelectric point (pI) of 6.13. This protein is predicted to localize in the nucleus and shares 88% amino acid identity with *Triticum urartu* WRKY49 protein (EMS52311.1) and 45% amino acid identity with the *Arabidopsis* IIc-type WRKY protein WRKY49 (NP_199143.1). We named this gene *TaWRKY49* (GenBank No. LC169122), the cDNA of which was 798-bp in length, and encodes a 258-amino-acid polypeptide with a molecular mass of 28.39 kDa and a theoretical pI of 5.83. The encoded protein, also predicted to localize in the nucleus, shares 95% amino acid identity with *Aegilops tauschii* WRKY62 protein (EMT18619.1) and 33% amino acid identity with the *Arabidopsis* III-type WRKY protein WRKY62 (NP_195810.2). Thus, the gene was named *TaWRKY62* (GenBank No. LC169123). *TaWRKY49* and *TaWRKY62* were mapped onto wheat chromosome 3B and the long arm of 5B, respectively. *TaWRKY49* was clustered in a large clade within the subgroup IIc of the WRKY family, with highest homology to *AtWRKY49*, while *TaWRKY62* belonged to a large clade of the group III WRKY members (Fig 1). Both TaWRKY49 and TaWRKY62 have conserved WRKY domains that includes one WRKYGQK sequence at the N-terminus. However, there is a C_2_-H_2_ (C-X_4_-C-X_23_-H-X_1_-H)-type zinc-finger motif at the C-terminus of TaWRKY49 (S2 Fig), and a C_2_-HC (C-X_7_-C-X_29_-H-X_1_-C)-type zinc-finger motif at the C-terminus of TaWRKY62 (S2 Fig). Based on these structural characteristics and the classification criteria [5], TaWRKY49 and TaWRKY62 were classified to the subgroup IIc and group III of WRKY families, respectively.

### Transcriptional changes of *TaWRKY49* and *TaWRKY62* during HTSP in response to *Pst*

**Fig 2:**
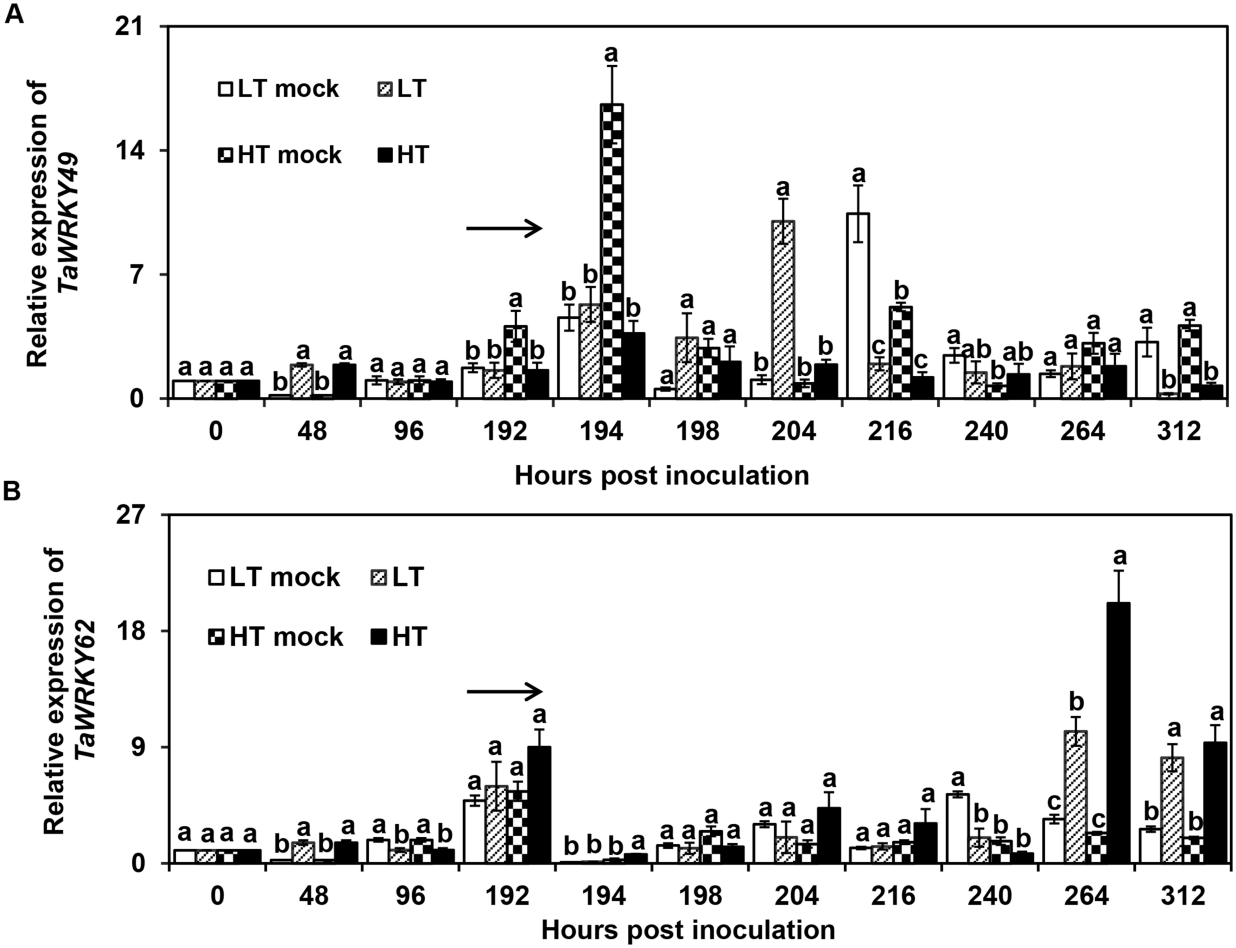
Expression levels of *TaWRKY49* and *TaWRKY62* exposed to high temperature after inoculation with *Puccinia striiformis* f. sp. *tritici*. Expression levels of (A) *TaWRKY49* and (B) *TaWRKY62* subjected to low temperature (LT) (constant 15°C) and high temperature (HT) [15°C for 192 h after *Pst* inoculation, then 20°C for 24 h, and back to 15°C after inoculation with *Puccinia striiformis* f. sp. *tritici* (*Pst*). LT Mock, low temperature treatment without inoculation of *Pst*; HT Mock, high temperature treatment without inoculation of *Pst*. The arrow indicates the beginning of the HT treatment. Relative gene expression levels were related to the level observed at 0 hpi. Three biological replicates were performed independently for each treatment. Error bars indicate standard error.

After the exposure to HT [beginning 192 h post inoculation (hpi) for 24 h], the expression of *TaWRKY49* was down-regulated at 194, 216 and 312 hpi (*P* < 0.05) when compared to the HT mock (HT but without *Pst* inoculation), and its expression level was lower (*P* < 0.05) than the low temperature *Pst* inoculation (LT) treatment at 204 hpi (Fig 2A). Under LT treatment, the expression level of *TaWRKY49* was higher than the LT mock (LT but without *Pst* inoculation) at 48 and 204 hpi, while thereafter, it was lower than the LT mock at 216 and 312 hpi (Fig 2A). The relative expression of *TaWRKY62* was elevated in the HT treatment in comparison with the HT mock at 48, 194, 264 and 312hpi, and was higher in the HT treatment than in the LT treatment at 264 hpi. In particular, at 264 hpi, the expression level of *TaWRKY62* was highest at HT treatment among the four treatments (Fig 2B).

### Phenotypes of *TaWRKY49-*silenced and *TaWRKY62*-silenced plants during the HTSP resistance to *Pst*

**Fig 3:**
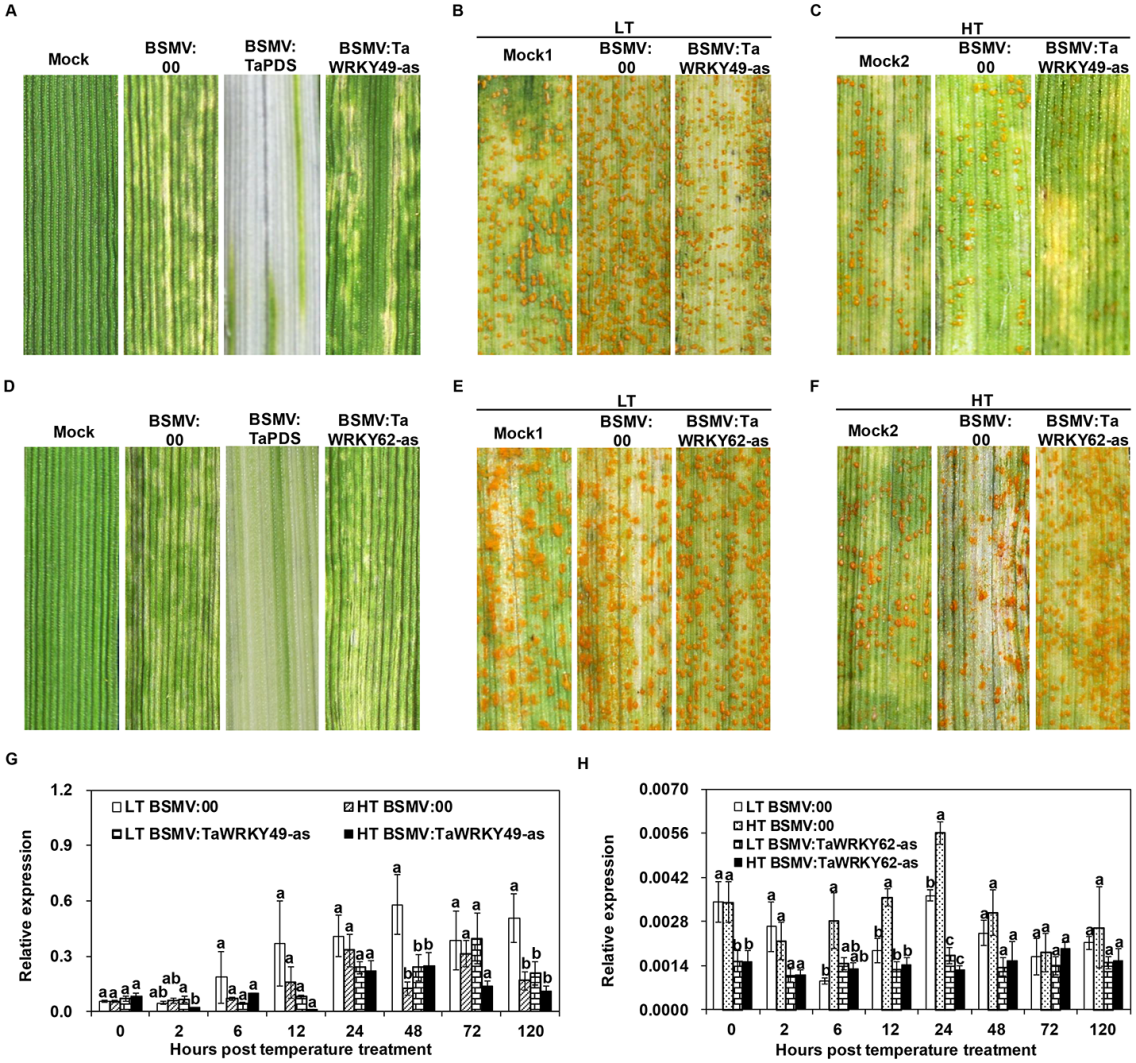
Phenotypes of *TaWRKY49*- and *TaWRKY62*-silenced leaves when inoculated with *Pst* subjected to low and high temperature. The phenotypes of (A, B and C) *TaWRKY49* or (D, E and F) *TaWRKY62*-silenced wheat leaves after *Puccinia striiformis* f. sp. *tritici* (*Pst*) inoculation at high temperature (HT) [15°C for the first 192 h post inoculation (hpi), then 20°C for 24 h, and back to 15°C] and low temperature (LT) (constant 15°C). (A, D) Mild chlorotic mosaic symptoms of BSMV at 9 days post-inoculation (dpi) (Mock: plants treated with FES buffer). Disease symptoms on the fourth leaves that were pre-inoculated with BSMV-derived RNAs, challenged with *Pst* race CYR32, and then subjected to (B, E) LT and (C, F) HT treatments. Disease symptoms were photographed on 14 dpi. Mock1 and Mock2: wheat plants were pre-inoculated with FES buffer, then inoculated with CYR32 and subjected to the LT and HT treatments, respectively. (G) Expression level of *TaWRKY49* in the fourth leaves of the plants that were pre-inoculated with BSMV: 00 or BSMV: TaWRKY49-as on the second leaves, followed by inoculation of *Pst* subjected to the HT or LT treatment. (H) Expression level of *TaWRKY62* in the fourth leaves of the plants that were pre-inoculated with BSMV: 00 or BSMV: TaWRKY62-as on the second leaves, followed by inoculation of *Pst* subjected to the HT or LT treatment. 0 hptt: 192 hours post-inoculation (hpi) of *Pst* from which HT was applied. Three biological replicates were performed independently for each treatment. Error bars indicate standard error.

*TaWRKY49* and *TaWRKY62* were silenced individually in wheat seedlings using the *Barley stripe mosaic virus* (BSMV)-induced gene-silencing (VIGS) system [26, 27]. The *phytoene desaturase* (*PDS*) gene was silenced by inoculation of the recombinant virus BSMV:TaPDS onto the surface of the second leaf of wheat seedlings at the two-leaf stage as the positive control. The empty vector (BSMV:00) was used as the negative control. At 9 days post-inoculation (dpi), photobleaching symptoms were observed for the BSMV:TaPDS inoculation (Fig 3A and 3D), and the typical striping mosaic symptoms were apparent on new leaves of plants for BSMV:00 inoculation (Fig 3A and 3D). The mock wheat plants (FES buffer inoculated) developed new normal leaves under the same conditions, suggesting that the specific silencing of *TaPDS* occurred on the BSMV:TaPDS-inoculated leaves (Fig 3A and 3D).

BSMV-inoculated plants displayed mild chlorotic mosaic symptoms at 9 dpi. At 14 dpi, the *TaWRKY49*-silenced (BSMV:TaWRKY49-as) leaves under the LT treatment showed fewer uredinia than the non-silenced (BSMV:00) (Fig 3B), and the typical HR phenotype was observed for the HT-treated *TaWRKY49*-silenced leaves, showing necrotic stripes with smaller pustules than the HT-treated non-silenced leaves (Fig 3C). Under the HT treatment, the *TaWRKY62*-silenced leaves exhibited stripes without chlorosis and abundant pustules. The leaves in which *TaWRKY62* was not silenced displayed necrotic/chlorotic stripes with limited sporulation (Fig 3F).

Compared with the BSMV:00 vector inoculated leaves, the expression level of *TaWRKY49* in silenced plants with BSMV carrying *TaWRKY49*-as was reduced (*P* < 0.01) for the LT treatment (S3 Fig) after inoculation with *Pst*. For the HT treatment, the transcriptional level of *TaWRKY49* in *TaWRKY49*-silenced and non-silenced leaves were reduced, though not significantly (Fig 3G). Similarly, the expression level of *TaWRKY62* in *TaWRKY62*-silenced leaves was reduced under the LT treatment (S3 Fig) after *Pst* inoculation. The levels were also reduced compared with the non-silenced leaves for HT treatment (*P*<0.05; Fig 3H), suggesting the silencing is effective under both temperature conditions.

### Changes in the fungal development and host responses in wheat leaves with *TaWRKY49* or *TaWRKY62* silenced

**Fig 4:**
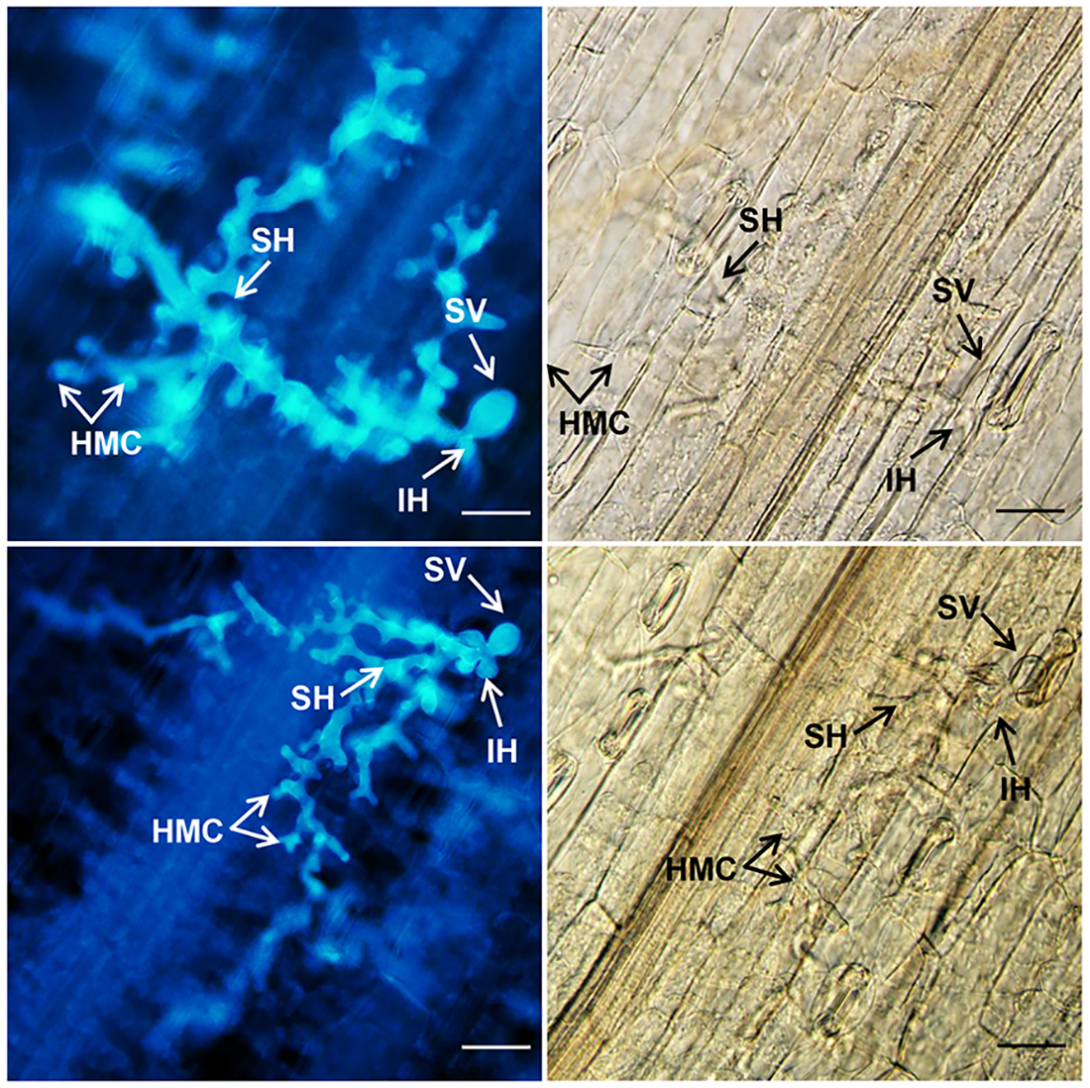
Histological observation of *Puccinia striiformis* f. sp. *tritici* (*Pst*) development in *TaWRKY49-*silenced leaves of Xiaoyan 6 at the low temperature (15°C) treatment. Photographs were obtained from BSMV:00-infected (top panels) and BSMV:TaWRKY49-as-infected (bottom panels) leaves inoculated with *Pst* race CYR32 under an epifluorescence (left panels) or light microscope (right panels) at 120 h post-inoculation (hpi). SV, substomatal vesicle; IH, initial hyphae; HMC, haustorial mother cell; SH, secondary hyphae. Scale bars = 100 μm.

The colony linear length and number of haustorial mother cells and host necrotic cells of *Pst* were assessed microscopically (Fig 4). At 120 hpi, hyphal length of *Pst* in *TaWRKY62*-silenced leaves was 58.13 μm more than those leaves where *TaWRKY62* was not silenced, while the number of *Pst* haustorial mother cells in *TaWRKY49*-silenced leaves were fewer than those observed in the non-silenced leaves (Table 1). Necrotic cells were rarely seen on the silenced leaves.

**Table 1:** Histological observations of *Puccinia striiformis* f. sp. *tritici* (*Pst*) development and host responses in *TaWRKY49*- and *TaWRKY62*-silenced wheat leaves under the low temperature (LT, 15°C) treatment.

Treatment^a^	Length of hyphae^b^(μm)		Number of haustorial mother cells^c^
	48 hpi	120 hpi	
**BSMV:00**	25.28±1.22^a^A	164.40±6.31^b^A	19.17±0.99^a^A
**BSMV:TaWRKY62-as** **BSMV:TaWRKY49-as**	26.61±0.90^a^ 24.83±0.73A	222.53±8.53^a^ 172.21±9.92A	19.81±0.81^a^ 16.50±0.88B

Lowercase letters indicate the comparison between BSMV:00 and BSMV:TaWRKY62-as, uppercase letters indicate the comparison between BSMV:00 and BSMV:TaWRKY49-as.

^a^ Wheat leaves pre-infected with BSMV:00 or recombinant BSMV:TaWRKY49-as or BSMV:TaWRKY62-as and then inoculated with *Pst* CYR32.

^b^ Average distance from the junction of the substomatal vesicle to hyphal tip (a half ellipse structure in which the polarity growth of hyphal is happened) (calculated from 30–50 infection sites).

^c^ Average number at an infection site (calculated from 30–50 infection sites).

hpi: hours post inoculation.

**Fig 5:**
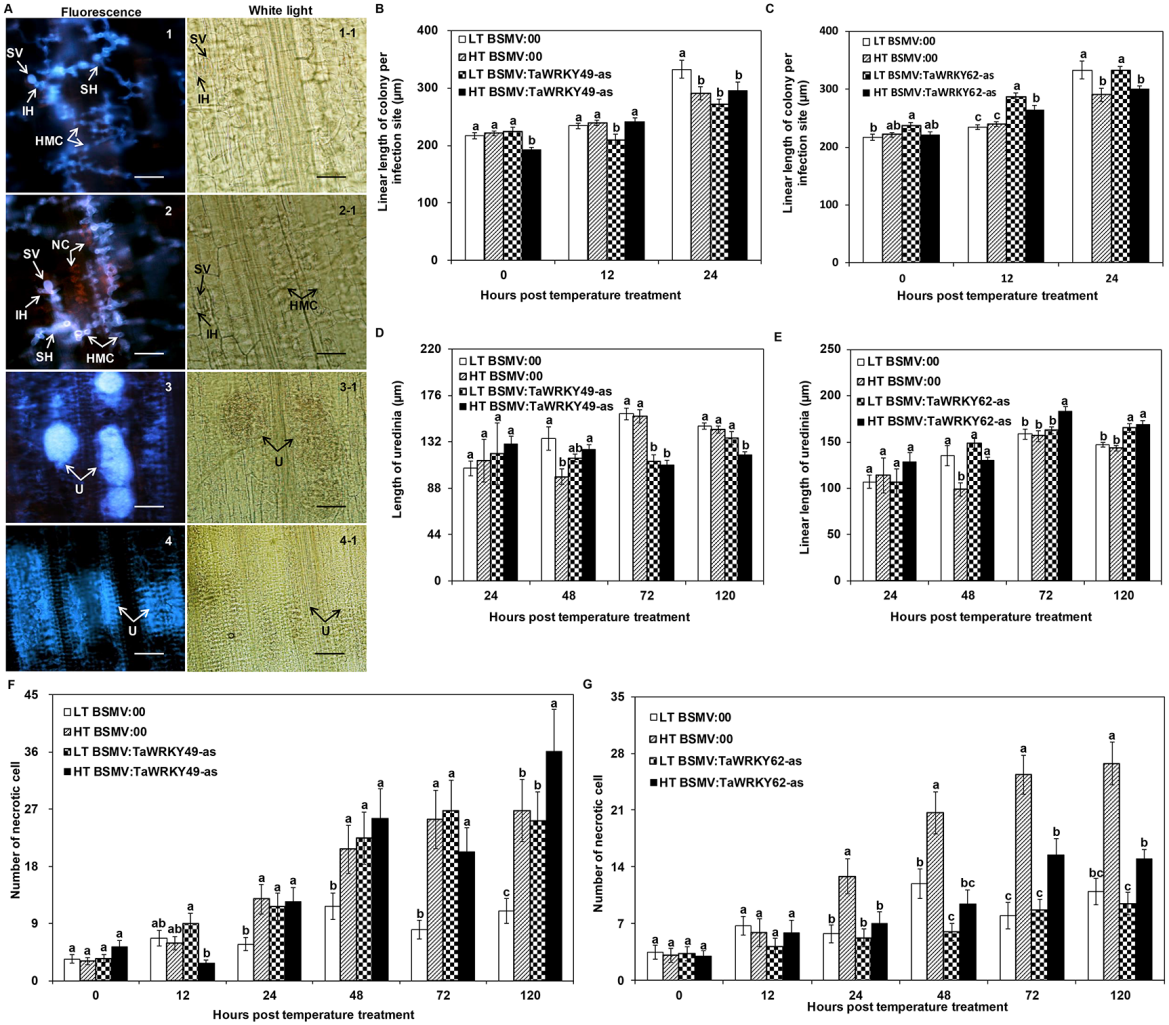
Histological observation of *TaWRKY49-* and *TaWRKY62*-silenced wheat leaves subjected to high temperatures after inoculation with *Pst*. (A) *Puccinia striiformis* f. sp. *tritici* (*Pst*) development in *TaWRKY49*-silenced leaves under the low temperature (LT) (constant 15°C) and high temperature (HT) [15°C for the first 192 h post-inoculation (hpi), then 20°C for 24 h, and back to 15°C] treatments: 1 = colony at LT, 2 = colony at HT, 3 = uredinia at LT, and 4 = uredinia at HT. SV = substomatal vesicle, IH = initial hyphae, HMC = haustorial mother cell, SH = secondary hyphae, NC = necrotic cell and U = uredinia. Scale bars = 100 μm. Photographs 1 and 2 were taken at 24 h post temperature treatment (hptt), while those of 3 and 4 were taken at 48 hptt. (B) Length of fungal colonies, (D) uredinium length and (F) number of necrotic cells from *TaWRKY49*-silenced leaves and from *TaWRKY62*-silenced leaves (C, E, G respectively) subjected to the LT and HT treatments after *Pst* inoculation. 0 hptt: 192 hours post inoculation (hpi), from which HT was applied. Error bars indicate standard error.

The length of the *Pst* colonies in infected wheat leaves was observed in response to the two different temperature regimes (Fig 5A1 and 5A2). For the HT treatment, the length of colonies in the BSMV:TaWRKY49-as leaves was similar to that in the BSMV:00 leaves, except at 0 hptt (Fig 5B). HT treated *TaWRKY62*-silenced leaves developed lengthier colonies (*P* < 0.05) than those in *TaWRKY62* non-silenced leaves at 12 hptt (Fig 5C). From 24 hptt onwards, the pustules gradually formed, and some uredinia with sparsely scattered urediniospores were seen in the HT-treated leaves (Fig 5A4); whereas urediospores in LT-treated leaves gathered closely (Fig 5A3). For the HT treatment, the length of uredinia in BSMV:TaWRKY49-as leaves was shorter than that in BSMV:00 leaves from 72 hptt onwards (*P*<0.05; Fig 5D). In contrast, the BSMV:TaWRKY62-as leaves developed lengthier uredinia than the BSMV:00 leaves from 48 hptt onwards (*P* < 0.05; Fig 5E). At 120 hptt, the number of dead cells in the HT-treated BSMV:TaWRKY49-as leaves was higher than the BSMV:00 leaves (Fig 5F). Fewer necrotic cells in the HT-treated BSMV:TaWRKY62-as leaves were observed than the BSMV:00 leaves at 24 hptt onwards (Fig 5G).

### Accumulation of reactive oxygen species (ROS) in *TaWRKY49*- and *TaWRKY62*-silenced wheat leaves

**Fig 6:**
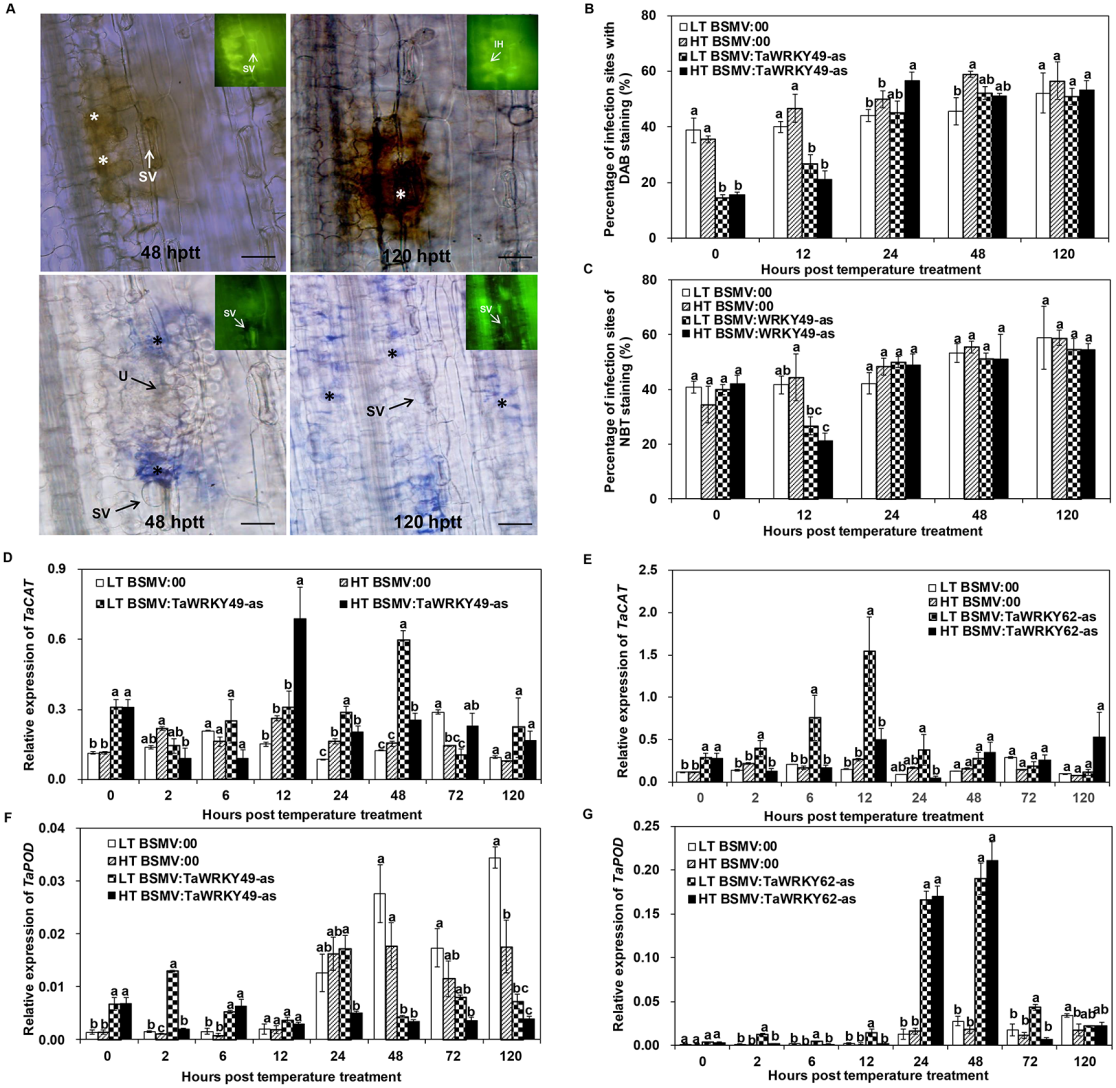
Detection of reactive oxygen species in gene-silenced leaves exposed to high temperature after *Pst* inoculation. (A) Histochemical localization of H_2_O_2_ (top panels) and O_2_^−^ (bottom panels) at the infection sites in BSMV:TaWRKY49-as-inoculated leaves subjected to the high temperature (HT) [15°C for the first 192 post-inoculation (hpi), then 20°C for 24 h, and back to 15°C] after inoculation with *Puccinia striiformis* f. sp. *tritici* (*Pst*). Photographs were obtained using light microscopy or epifluorescence after the HT treatment. * = guard cells of stoma harboring substomatal vesicle or mesophyll cells in contact with hyphae showing reddish-brown (H_2_O_2_ accumulation) or blue (O_2_^−^ accumulation) staining, SV = substomatal vesicle, IH = infection hyphae, U = uredinia. Bars = 100 μm. Percentages of infection sites exhibiting accumulation of (B) H_2_O_2_ and (C) O_2_^−^ in the *TaWRKY49*-silenced leaves when exposed to the HT and low temperature (LT) (constant 15°C) after inoculation with *Pst*. Relative expression of catalase (CAT) and peroxidase (POD) (D, F) *TaWRKY49*-silenced and (E, G) *TaWRKY62*-silenced leaves exposed to LT and HT after *Pst* inoculation. 0 hptt: 192 hours post inoculation (hpi) from which HT was applied. Three biological replicates were performed independently for each treatment. Error bars indicate standard error.

Accumulation of H_2_O_2_ was induced in the mesophyll cells or the cell walls of the leaves, which was evident by the reddish-brown staining due to 3, 3-diaminobenzidine (DAB) polymerization (Fig 6A). Under the HT treatment, the percentage point of DAB staining in the BSMV:TaWRKY49-as leaves was 20% and 25% lower than that of BSMV:00 leaves at 0 and 12 hptt, but higher at 24 hptt (Fig 6B). DAB staining was apparent at more than 50% of the infection sites at 24 hptt for TaWRKY49-silenced leaves under the HT (Fig 6B), indicating the importance of BSMV:TaWRKY49-as in reactions to *Pst* development at 24 h. There were no significant differences in the accumulation of H_2_O_2_ between *TaWRKY62*-silenced and non-silenced leaves under the HT condition (S4 Fig). Under the HT treatment, O_2_^−^ production levels were similar between *TaWRKY49*-silenced and non-silenced leaves (Fig 6C). There were no significant differences between *TaWRKY62*-silenced and non-silenced leaves in the percentage of infection sites with NBT staining under the HT treatment (S4 Fig).

Under the HT treatment, the expression level of *TaCAT* (catalase) in the infected leaves of BSMV:TaWRKY49-as was higher than in the BSMV:00 leaves at 0, 12 and 48 hptt (Fig 6D). In contrast, the expression of *TaCAT* did not differ between BSMV:TaWRKY62-as and BSMV:00 (Fig 6E). Under the HT treatment, the *TaPOD* (peroxidase) gene was induced in BSMV:TaWRKY49-as leaves before 12 hptt when compared with the BSMV:00 leaves. *TaPOD* was suppressed in the BSMV:TaWRKY49-as leaves, when compared to the BSMV:00 leaves at 24 hptt (Fig 6F). The expression level of *TaPOD* in the HT-treated *TaWRKY62*-silenced leaves was higher than the HT-treated *TaWRKY62* non-silenced leaves at 24 and 48 hptt (Fig 6G).

**Fig 7:**
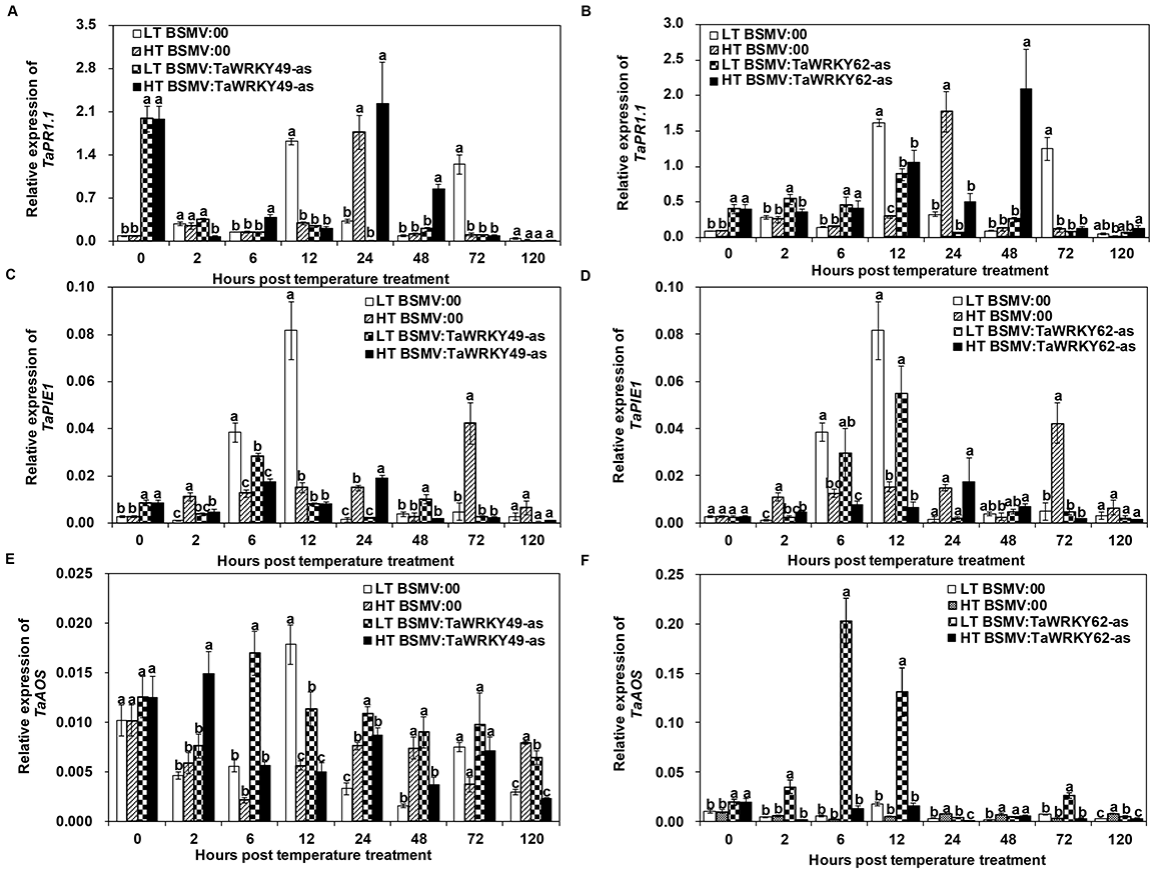
Relative expression analyses of defense-related genes in *TaWRKY49*- or *TaWRKY62*-silenced Xiaoyan 6 leaves of subjected to high temperature after inoculation with *Puccinia striiformis* f. sp. *tritici* (*Pst*). A-F shows the expression level of pathogenesis-related (*TaPR1*.*1*), pathogen-induced ethylene response factor 1 (*TaPIE1*) and allene oxide synthase (*TaAOS*) genes in *TaWRKY49*-silenced or *TaWRKY62*-silenced leaves when exposed to the high temperature (HT) [15°C for the first 192 h post-inoculation (hpi), then 20°C for 24 h, and back to 15°C] and low temperature (LT) (constant 15°C) after inoculation with *Pst*. 0 hptt: 192 hours post inoculation (hpi) from which HT was applied. Three biological replicates were performed independently for each treatment. Error bars represent standard error.

Under the HT treatment, the expression level of a SA marker gene (*TaPR1*.*1*) in *TaWRKY49*-silenced leaves was higher than in the non-silenced leaves at 0, 6 and 48 hptt (Fig 7A). Similarly in the HT treatment, the level of *TaPR1*.*1* expression in *TaWRKY62*-silenced leaves was higher than in the non-silenced leaves at 0, 6, 12 and 48 hptt (Fig 7B). The expression of pathogen-induced ethylene response factor 1 (*TaPIE1*), an ET-responsive gene, was reduced in the *TaWRKY49*-silenced leaves (Fig 7C) and in the *TaWRKY62*-silenced leaves (Fig 7D) compared with the non-silenced leaves under the HT treatment. The marker gene of JA signaling, allene oxide synthase (*TaAOS*), was induced rapidly from the *TaWRKY49*-silenced leaves in HT treatment at 2 hptt when compared to the HT-treated non-silenced (Fig 7E). The expression level of *TaAOS* did not differ between the HT-treated *TaWRKY62*-silenced and non-silenced leaves at earlier stage (Fig 7F); however, at the later stage, the level of *TaAOS* expression was lower in *TaWRKY62*-silenced leaves than in the non-silenced leaves at 24 and 120 hptt (Fig 7F).

### Expression levels of *TaWRKY49* and *TaWRKY62* in response to abiotic stress and in different wheat tissues

*TaWRKY49* was up-regulated on exogenous application of ET, ABA and SA (Table 2). Under the ET treatment, this gene was rapidly induced within 0.5 h post-treatment (hpt), and its expression level reached the peak at 6 hpt (the peak was about 15-fold higher than the control at 0 hpt) (Table 2). The induction of *TaWRKY49* by the application of SA and ABA only occurred at 0.5 and 2 hpt, respectively (Table 2). In contrast, methyl jasmonate (MeJA) application did not affect the expression of *TaWRKY49*, and H_2_O_2_ treatment reduced the expression of *TaWRKY49* (Table 2). *TaWRKY62* was up-regulated by all five hormone treatments (Table 2). Under ABA and ET treatments individually, the peak of the expression level of *TaWRKY62* reached was at 2 hpt (Table 2). Under H_2_O_2_, MeJA and SA treatments individually, this gene was fastly induced and its expression level reached at a peak at 0.5 hpt (Table 2).

**Table 2:** Expression patterns of *TaWRKY49* and *TaWRKY62* under different abiotic stresses and in different wheat tissues.

Gene		Phytohormone					Threshold Temperatures		Root	Stem	Leaf
	hpt^a^	ABA^b^	ET^c^	H_2_O_2_^d^	MeJA^e^	SA^f^	4°C	40°C			
***TaWRKY49***	0	1.00±0.00^b^	1.00±0.00^e^	1.00±0.00^a^	1.00±0.00^a^	1.00±0.00^b^	1.00±0.00^d^	1.00±0.00^a^	0.93±0.01^b^	1.33±0.10^a^	1.00±0.00^b^
	0.5	0.77±0.13^b^	7.30±0.52^c^^d^	0.22±0.01^c^	0.53±0.02^a^	11.42±0.22^a^	-	-			
	2	3.18±0.07^a^	10.94±0.95^b^	0.68±0.08^b^	0.95±0.18^a^	0.49±0.06^b^	13.12±1.66^b^	0.22±0.03^b^			
	6	0.80±0.25^b^	15.40±1.45^a^	0.47±0.09^b^	0.86±0.20^a^	1.23±0.26^b^	24.33±1.51^a^	0.33±0.08^b^			
	12	0.71±0.17^b^	9.64±0.61^b^^c^	0.18±0.06^c^	0.64±0.17^a^	1.12±0.29^b^	24.08±1.75^a^	0.28±0.07^b^			
	24	1.04±0.10^b^	4.64±0.10^d^	0.21±0.03^c^	0.98±0.25^a^	0.55±0.05^b^	6.57±0.41^c^	0.31±0.08^b^			
***TaWRKY62***	0	1.00±0.00^b^^c^	1.00±0.00^c^	1.00±0.00^b^	1.00±0.00^b^	1.00±0.00^b^^c^	1.00±0.00^c^	1.00±0.00^a^	7.53±0.29^a^	0.54±0.06^b^	1.00±0.00^b^
	0.5	1.16±0.19^b^^c^	4.14±0.22^a^	1.49±0.26^a^	1.55±0.22^a^	4.97±0.59^a^	-	-			
	2	4.30±0.53^a^	4.31±0.56^a^	0.86±0.20^b^	0.77±0.07^b^	0.90±0.16^b^^c^	0.78±0.20^c^	0.93±0.16^a^			
	6	1.60±0.29^b^	2.97±0.41^b^	0.63±0.07^b^^c^	0.38±0.14^c^	1.60±0.34^b^	1.87±0.11^b^	0.41±0.04^b^			
	12	0.50±0.11^c^	1.02±0.10^c^	0.37±0.08^c^	0.37±0.07^c^	0.32±0.11^c^	1.50±0.13^b^^c^	0.75±0.14^a^			
	24	0.42±0.04^c^	1.59±0.12^c^	0.30±0.08^c^	0.80±0.07^b^	0.41±0.15^c^	4.82±0.52^a^	0.43±0.03^b^			

The expression levels were determined through RT-qPCR analyses and were relative to the levels observed at 0 hpi. Three biological replicates were performed independently for each treatment.

^a^ Hours post treatment,

^b^ abscisic acid,

^c^ ethylene,

^d^ hydrogen peroxide,

^e^ methyl jasmonate and

^f^ salicylic acid.

With regard to the extreme temperature treatments, the transcript levels of *TaWRKY49* and *TaWRKY62* were up-regulated by cold (4°C) stress and reached the peak at 6 and 24 hpt respectively (Table 2). And the transcript levels of both genes were down-regulated in response to heat (40°C) stress (Table 2). *TaWRKY49* was expressed predominantly in stem, but *TaWRKY62* was expressed predominantly in roots (Table 2).

## Discussion

WRKY TFs have been implicated in many plant defense processess including responses to biotic and abiotic stresses [5]. Many WRKY genes are responsive to infections, including those initiated by fungi [14, 32], bacteria [33, 34], and a virus [35], suggesting that WRKY TFs play critical roles in plant defense responses to pathogens. In this present study, in wheat HTSP resistance against *Pst*, *TaWRKY49* and *TaWRKY62* were down- and up-regulated, respectively. Inducing of silencing constructs targeting *TaWRKY49* led to the enhanced HTSP resistance. In contrast, the silencing of *TaWRKY62* led to reduced HTSP resistance. These results suggest a negative regulatory role of *TaWRKY49* TF and a positive regulatory role of *TaWRKY62* TF in wheat HTSP resistance against *Pst*.

Many WRKY TFs, such as *AtWRKY4* [36], *AtWRKY8* [37], *AtWRKY27* [38] and *AtWRKY48* [39] in *Arabidopsis*; *HvWRKY1* and *HvWRKY2* in barley [40]; *CaWRKY1* [41] and *CaWRKY58* [34] in pepper; and *OsWRKY62* in rice [16], function as negative regulators of disease resistance in plant-pathogen interactions. In the present study, the group-IIc WRKY gene, *TaWRKY49*, negatively regulated wheat HTSP resistance against *Pst*. In contrast, *TaWRKY62* functioned as a positive regulator in the wheat resistance. Thus, the two WRKY TFs affected plant resistance in opposite ways, and this may be due to the distinct roles played by the two WRKY proteins in regulating the crosstalk between defense signaling pathways. This may require that these WRKY TFs act as transcriptional activators or repressors in a gene-specific manner. Furthermore, the WRKY TFs could have opposite effects against different types of pathogens. For example, loss-of-WRKY70 function in *Arabidopsis* increased plant susceptibility to *Pseudomonas syringae*, *Erwinia carotovora*, *E*. *cichoracearum* and *Botrytis cinerea*, but increased the resistance to *A*. *brassicicola* [14, 42, 43]. Overexpression of *OsWRKY62* in rice compromises the resistance against *Xanthomonas oryzea* pv. *oryzea* [16]; while in this study, the silencing of *TaWRKY62* compromised HTSP resistance to *Pst*.

High temperature stimuli induced HTSP resistance to *Pst*, and hence the expression levels of relevant WRKY TFs were responsive in high temperature treatment. The molecular basis of the effects of high temperature on *TaWRKY49* and *TaWRKY62* in response to *Pst* is unknown, although *Pst* resistance genes affected by temperature have been reviewed recently [44]. In leaf rust resistance testing, the Thatcher wheat line with *Lr23* was susceptible to all isolates at all temperatures except for one isolate which was avirulent at 30°C and 10~30°C, indicating temperature specificity is necessary for wheat-leaf rust genetic interaction for *Lr23* in some cases [45]. During adult plant resistance to leaf rust on wheat cv. Thatcher (carries the *Lr22b* gene, and its nearisogenic lines *Lr34* and *Lr37*), *Lr22b* is inefficient; while *Lr34* slows down the disease development at a mean daily temperature below 16°C, but is poorly efficient at temperatures above 20°C; *Lr37* provides high resistance under all conditions [46], showing that some resistance genes were correlated with the temperature-dependent resistance. Two quantitative trait loci (QTLs) were detected from a population of 188 F_2:3_ families (from wheat cross Fundulea 900/‘Thatcher’), and the two QTLs were designated as QLr.hebau-1BL that was *Lr46* and QLr.hebau-7DS that was *Lr34*, respectively [47]. These genes have minor effects, conferring partial, durable resistance to leaf rust [48, 49]. Therefore, the character of *TaWRKY49*/*TaWRKY62* in HTSP resistance to *Pst* is similar with above genes-temperature sensitive and exerting minor effect. *Yr36* functions as a regulatory gene, and its START domain is postulated to bind lipids from *Pst* at high temperature and change its conformation, which might cause the kinase domain to initiate a signaling cascade leading to programmed cell death [50]. In rice, OsWRKY62 interacts with *Xa21*-cleaved intracellular domain exclusively in the nucleus, mediating immune responses [51]. Although OsWRKY62 itself is localized in an unknown intracellular structure/organelle, OsWRKY62 and OsWRKY76 can form a hetero-complex in the nucleus, suggesting that these two TFs may function collaboratively [52]. TaWRKY62 at high temperature may either interact with other proteins or directly regulate the transcriptional reprogramming of defense-related genes. Negative regulators of disease resistance, such as AtWRKY17, AtWRKY11 [53], CaWRKY1 [41], HvWRKY1/2 [40] and OsWRKY62 [16], are inferred to prevent the inappropriate activation of defense responses at suboptimal concentrations of signal molecules, or to turn off the activated defense reaction generated by positive regulators once the pathogen infection has been halted, since the defense responses against disease-causing microbes are energy consuming processes [54]. Thus, the negative role of *TaWRKY49* in HTSP resistance against *Pst* may lie in preventing the inappropriate activation or in turning off excessive defense responses.

WRKY TFs are nodes for cross-talk between SA, JA and ET signalling pathways and involved in plant defense through these signalling parhways [14, 55, 56]. Crosstalk among SA, JA and ET has emerged as an important regulation switch in plant disease resistance [57, 58]. Usually, SA signaling is specific to resistance responses against biotrophic pathogens, whereas JA/ET signaling against necrotrophic pathogens [59, 60]. Negative regulators of disease resistance, such as AtWRKY17, AtWRKY11 [48], CaWRKY1 [41], HvWRKY1/2 [40] and OsWRKY62 [16], are transcriptionally induced in response to pathogen infection, as well as in response to SA or JA [16, 17, 40, 41, 48]. However, the expression of *TaWRKY49* was down-regulated by *Pst* infection in the HT treatment, up-regulated by ET and SA and was not affected by MeJA. We speculate that the relatively higher temperatures may change the transcription level of *TaWRKY49* that is induced by SA and ET signaling. In *Arabidopsis*, systemic acquired resistance (SAR) is strongly correlated with the coordinate expression of a set of genes encoding proteins that include the pathogenesis-related (PR) proteins, including PR1-1a [61, 62]. In this study, *TaPR1*.*1* was induced in both *TaWRKY49*- and *TaWRKY62*-silenced wheat leaves under the HT treatment. These results suggest that the increased disease resistance of silenced-*TaWRKY49* and increased susceptibility of silenced-*TaWRKY62* wheat leaves at high temperature may partially depend on a common SA signaling pathway that is invovled in SAR.

The ROS network is essential for the induction of disease resistance [63, 64]. H_2_O_2_ is one of the primary ROS species at the site of pathogen infection [65]. In addition to being a direct protective agent, the oxidative burst functions as a threshold trigger for hypersensitive cell death [11, 66–68]. ROS-induced HR can act as a defense reaction against pathogens [69, 70]. In HT-treated *TaWRKY49*-silenced leaves after *Pst* inoculation, H_2_O_2_ and O_2_^–^ were produced rapidly from 24 hptt and the accumulation level of these chemicals was higher than the HT-treated non-silenced, accompanied by the rapid induction of antioxidant enzyme gene *TaPOD*. There was a positive correlation between POD and disease resistance in plants: after inoculation of pathogen, the POD activity was rapidly increased in a resistant cultivar but not enhanced or delayed in a susceptible cultivar [71]. The expression of *TaPOD* was induced early in the *TaWRKY49*-silenced leaves at the beginning of HT treatment, which might contribute to the induction of disease resistance. In the earlier stage of ROS production, that was induced by pathogen attacks, CAT can decompose H_2_O_2_ into O_2_^–^, acting to trigger benzoic acid to form SA and leading to systemic acquired resistance (SAR) [72]. Thus, it is reasonable to speculate that up-regulation of *TaCAT* in the HT-treated silenced-*TaWRKY49* leaves may play a role in triggering SA signaling. In contrast, in the HT-treated *TaWRKY62*-silenced leaves, the *TaCAT* expression level did not vary noticeably, and *TaPOD* was induced only at the later stage (after 12 hptt), which is consistent with the increased susceptibility at high temperature due to silencing *TaWRKY62*. These results suggested that ROS signaling is likely involved in the WRKY gene-regulated HTSP resistance to *Pst* via independently or interacting with other signaling pathways. The overexpression of a canola MYB gene-*BnaMYB78* in *Nicotiana benthamiana* modulates ROS-dependent cell death through regulating the transcription of a few ROS- and defense-related genes [73]. In addition, colony and uredinium formation was also delayed, and the number of necrotic cell, representing the level of HR [74], was increased significantly in HT-treated *TaWRKY49*-silenced wheat leaves. Thus, *TaWRKY49* negatively regulates the HTSP resistance to *Pst* partially via ROS signaling dependent HR cell death. On the other hand, the accumulation of H2O2 and O_2_^–^ in HT-treated *TaWRKY62*-silenced leaves after *Pst* infection did not vary significantly. The longer uredinium length and fewer necrotic cells were observed in HT-treated *TaWRKY62*-silenced leaves, suggesting that *TaWRKY62* positively participates in the HTSP resistance probably through enhancement of cell death independent of ROS. Although ROS production is an essential component for the onset of local cell death in micro-hypersensitive response and induction of host resistance [75], it may not be the only factor that leads to cell death. Further research is needed to understand the relationship between ROS and cell death.

**Fig 8:**
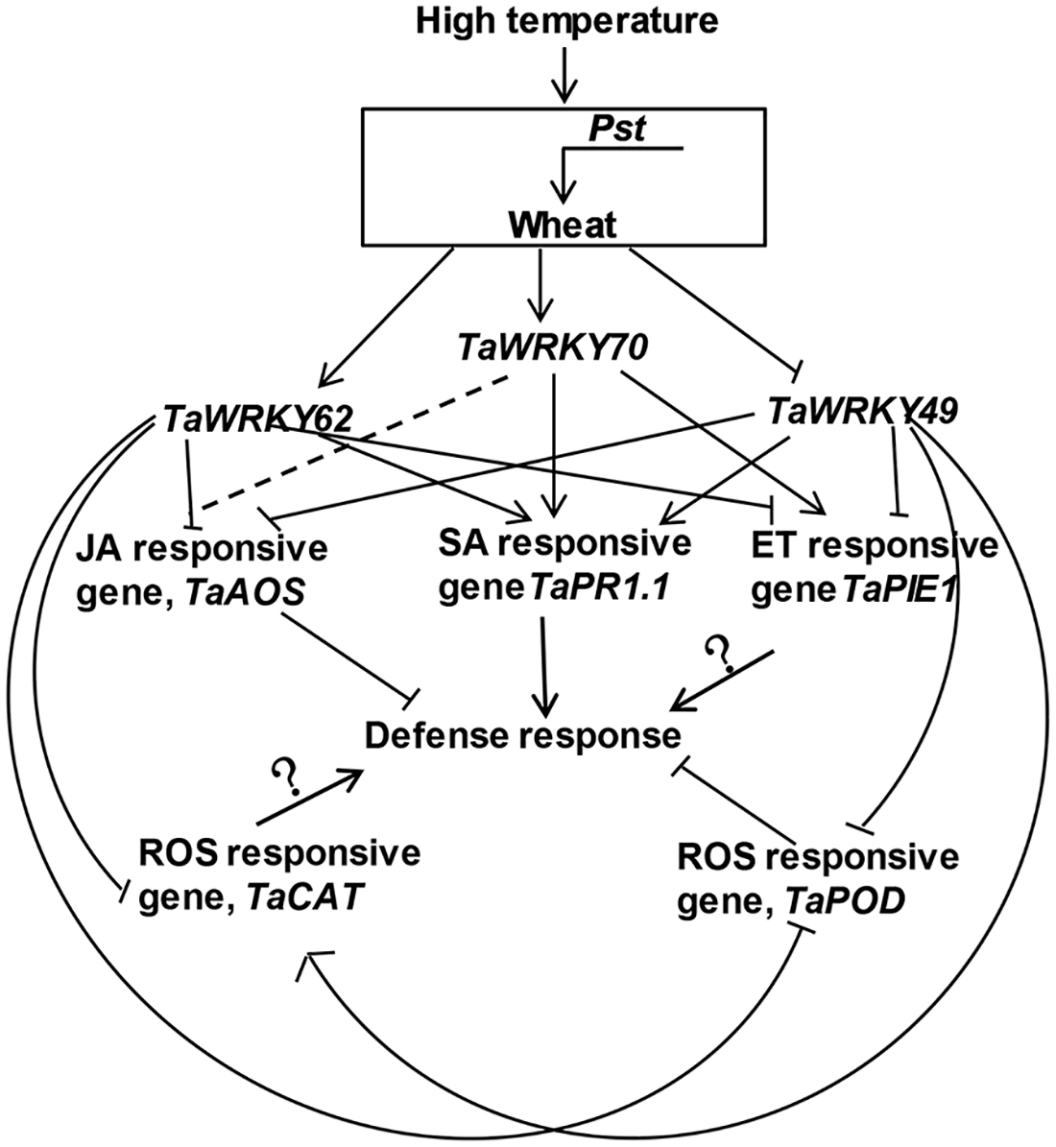
Model showing WRKY-mediated regulation of plant defense responses and signalings in high-temperature induced resistance to *Pst*. The solid line with an arrow denotes enhancement; solid line without an arrow denotes suppression; solid line with an arrow and a symbol “?” denotes unknown; and the dotted lines denote no effect. *Pst*, *Puccinia striiformis* f. sp. *tritici*; JA, jasmonic acid; H_2_O_2_, hydrogen; SA, salicylic acid; ET, ethylene; and ROS, reactive oxygen species.

In response to multiple stresses, the wheat WRKY genes exhibited similar responses. For example, they were induced by multiple phytohormones and cold stress (4°C), and repressed by heat stress (40°C). We recently showed that *TaWRKY70* positively regulates the HTSP resistance to *Pst* via enhancing the expression of both *TaPR1*.*1* and *TaPIE1* genes [15]. These results together indicate that SA-, ET- and JA-related genes as well as ROS-related genes could be differentially regulated by *TaWRKY49*, *TaWRKY62* and *TaWRKY70* during the wheat HTSP resistance against *Pst* infection (Fig 8). Further understanding on the crosstalk between phytohormone- and ROS-mediated signaling will provide new insights into how these WRKY TFs regulate HTSP resistance against *Pst*.

## Supporting information

S1 Table The primer sequences used in this study. (DOC)

S2 Table GenBank accession numbers of the WRKY proteins used for generating the dendrogram and alignments. Gh: *Gossypium hirsutum*; Tu: *Triticum urartu*; Hv: *Hordeum vulgare*; Hvpp: *Hordeum vulgare* predicted protein; Os: *Oryza sativa* Indica Group; SbSORBIDRAFT_03g039550: *Sorghum bicolor* hypothetical protein SORBIDRAFT_03g039550; PtPOPTR_0006s08730g: *Populus trichocarpa* hypothetical protein POPTR_0006s08730g; Tc: *Theobroma cacao*; Ta: *Triticum aestivum*; At: *Arabidopsis thaliana*; Aet: *Aegilops tauschii*; Mt: *Medicago truncatula*; Me: *Manihot esculenta*; OSJ: *Oryza sativa* Japonica Group. Gh: *Gossypium hirsutum*; Tu: *Triticum urartu*; Hv: *Hordeum vulgare*; Hvpp: *Hordeum vulgare* predicted protein; Os: *Oryza sativa* Indica Group; SbSORBIDRAFT_03g039550: *Sorghum bicolor* hypothetical protein SORBIDRAFT_03g039550; PtPOPTR_0006s08730g: *Populus trichocarpa* hypothetical protein POPTR_0006s08730g; Tc: *Theobroma cacao*; Ta: *Triticum aestivum*; At: *Arabidopsis thaliana*.
(DOC)

S3 Table The raw data of the tables and figures related to this paper. (XLS)

S1 Fig Specificity test of amplicons via reverse transcription-quantitative polymerase chain reaction (RT-qPCR) and the efficiency (E) of the primer-specific polymerase chain reaction (PCR) amplification. (A) Agarose gel (2%) electrophoresis showing amplification of a single product of the expected size for all tested genes. M represents the DL2000 DNA marker. (B) Dissociation curves with single peaks generated for all genes. (C) The efficiency of primer-specific polymerase chain reaction (PCR) amplifications.
(PDF)

S2 Fig The alignment of (A) TaWRKY49 and (B) TaWRKY62 with their top 9 homologous. The WRKYGQK peptide stretch is shown in red. The zinc-finger-like motifs in the domains are shown in yellow, the GenBank accession numbers were given in S2 Table.
(PDF)

S3 Fig Efficiency of silencing (A) *TaWRKY49* and (B) *TaWRKY62* by virus-induced gene silencing under the low temperature treatment after inoculation with *Puccinia striiformis* f. sp. *tritici*. **(Student’s *t*-test, *P* < 0.01) or *(Student’s *t*-test, *P* < 0.05) indicate significant differences in the mean of gene expression level between the BSMV: WRKY49/62-as-inoculated plants and the BSMV: 00-inoculated plants. Error bars indicate standard error.
(TIF)

S4 Fig Detection of reactive oxygen species (ROS) in the *TaWRKY62*-silenced leaves in exposure to high temperature (HT) [15°C initially, then 20°C from 192 h post-inoculation (hpi) for 24 h, and finally back to 15°C again) and low temperature (LT) (constant 15°C) after infection of *Puccinia striiformis* f. sp. *tritici* (*Pst*). Percentages of infection sites exhibiting (A) H_2_O_2_ and (B) O_2_^−^ accumulation in *TaWRKY62*-silenced leaves in exposure to HT and LT after inoculation with *Pst*. 0 hptt: 192 hours post inoculation (hpi) from which HT was applied. Error bars indicate standard error.
(TIF)

## References

[1] Dangl JL, Horvath DM, Staskawicz BJ. Pivoting the plant immune system from dissection to deployment. Science. 2013; 341(6147): 746–751. doi: 10.1126/science.1236011 23950531 10.1126/science.1236011 PMC3869199

[2] Yang Y, Shah J, Klessig DF. Signal perception and transduction in plant defense responses. Genes Dev. 1997; 11(13): 1621–1639. 9224713 10.1101/gad.11.13.1621

[3] Rushton PJ, Somssich IE. Transcriptional control of plant genes responsive to pathogens. Curr Opin Plant Biol. 1998; 1(4): 311–315. 10066598 10.1016/1369-5266(88)80052-9

[4] Eulgem T, Weigman VJ, Chang HS, McDowell JM, Holub EB, Glazebrook J, et al Gene expression signatures from three genetically separable resistance gene signaling pathways for downy mildew resistance. Plant Physiol. 2004; 135(2): 1129–1144. doi: 10.1104/pp.104.040444 15181204 10.1104/pp.104.040444 PMC514145

[5] Eulgem T, Rushton PJ, Robatzek S, Somssich IE. The WRKY superfamily of plant transcription factors. Trends Plant Sci. 2000; 5(5): 199–206. 10785665 10.1016/s1360-1385(00)01600-9

[6] Eulgem T, Somssich IE. Networks of WRKY transcription factors in defense signaling. Curr Opin Plant Biol. 2007; 10(4): 366–371. doi: 10.1016/j.pbi.2007.04.020 17644023 10.1016/j.pbi.2007.04.020

[7] Zhang Y, Wang L. The WRKY transcription factor superfamily: its origin in eukaryotes and expansion in plants. BMC Evol Biol. 2005; 5(1): 1. 15629062 10.1186/1471-2148-5-1 PMC544883

[8] Okay S, Derelli E, Unver T. Transcriptome-wide identification of bread wheat WRKY transcription factors in response to drought stress. Mol Genet Genomics. 2014; 289(5): 765–781. doi: 10.1007/s00438-014-0849-x 24748053 10.1007/s00438-014-0849-x

[9] Satapathy L, Singh D, Ranjan P, Kumar D, Kumar M, Prabhu KV, et al Transcriptome-wide analysis of WRKY transcription factors in wheat and their leaf rust responsive expression profiling. Mol Genet Genomics. 2014; 289(6): 1289–1306. doi: 10.1007/s00438-014-0890-9 25098419 10.1007/s00438-014-0890-9

[10] Menke FLH, Kang HG, Chen ZX, Park JM, Kumar D, Klessig DF. Tobacco transcription factor WRKY1 is phosphorylated by the MAP kinase SIPK and mediates HR-like cell death in tobacco. Mol Plant-Microbe Interac. 2005; 18(10): 1027–1034. 10.1094/MPMI-18-1027 16255241

[11] Lamb C, Dixon RA. The oxidative burst in plant disease resistance. Annu Rev Plant Physiol Plant. Mol Biol. 1997; 48(1): 251–275. 15012264 10.1146/annurev.arplant.48.1.251

[12] Torres MA, Jones JD, Dangl JL. Reactive oxygen species signaling in response to pathogens. Plant Physiol. 2006; 141(2): 373–378. doi: 10.1104/pp.106.079467 16760490 10.1104/pp.106.079467 PMC1475467

[13] Gregersen PL, Thordal-Christensen H, Forster H, Collinge DB. Differential gene transcript accumulation in barley leaf epidermis and mesophyll in response to attack by *Blumeria graminis* f. sp. *hordei* (syn. *Erysiphe graminis* f. sp. *hordei*). Physiol Mol Plant Pathol. 1997; 51(2): 85–97.

[14] Li J, Brader G, Kariola T, Palva ET. WRKY70 modulates the selection of signaling pathways in plant defense. Plant J. 2006; 46(3): 477–491. doi: 10.1111/j.1365-313X.2006.02712.x 16623907 10.1111/j.1365-313X.2006.02712.x

[15] Wang J, Tao F, An F, Zou Y, Tian W, Chen X, et al Wheat transcription factor TaWRKY70 is positively involved in high-temperature seedling-plant resistance to *Puccinia striiformis* f. sp. *tritici*. Mol Plant Pathol. 2016; doi: 10.1111/mpp.12425 27145738 10.1111/mpp.12425 PMC6638234

[16] Peng Y, Bartley LE, Chen X, Dardick C, Chern M, Ruan R, et al OsWRKY62 is a negative regulator of basal and Xa21-mediated defense against *Xanthomonas oryzae* pv. *oryzae* in rice. Mol Plant. 2008; 1(3): 446–458. doi: 10.1093/mp/ssn024 19825552 10.1093/mp/ssn024

[17] Chen XM. Epidemiology and control of stripe rust [*Puccinia striiformis* f. sp *tritici*] on wheat. Can J Plant Pathol. 2005; 27(3): 314–337.

[18] Villareal LM, Lannou C, De Vallavieille-Pope C, Neema C. Genetic variability in *Puccinia striiformis* f. sp. *tritici* populations sampled on a local scale during natural epidemics. Appl Environ Microbiol. 2002; 68(12): 6138–6145. doi: 10.1128/AEM.68.12.6138-6145.2002 12450838 10.1128/AEM.68.12.6138-6145.2002 PMC134396

[19] Chen XM, Line RF. Gene-action in wheat cultivars for durable, high-temperature, adult-plant resistance and interaction with race-specific, seedling resistance to *Puccinia*-*striiformis*. Phytopathology. 1995; 85(5): 567–572.

[20] Qayoum A, Line RF. High-temperature, adult-plant resistance to stripe rust of wheat. Phytopathology. 1985; 75(10): 1121–1125.

[21] An F, Tao F, Wang J, Tian W, Shang H, Hu X. Optimal conditions of expression of high-temperature resistance to stripe rust in Xiaoyan 6. J Triticeae Crops. 2015; 35(9): 1314–1319.

[22] Ma Q, Shang HS. High-temperature resistance of wheat cultivar Xiaoyan series to wheat stripe rust. Acta Agric Bor-Occid Sin. 2000; 9(1): 39–42.

[23] Shang HS. High temperature resistance of wheat to stripe rust. Sci Agric Sin. 1998; 31(4): 46–50.

[24] Shang HS, Wang LG, Lu HP, Jing JX. Characteristics of expression of high-temperature resistance to stripe rust in wheat. Acta Phytophy Sin. 1997; 24: 97–100.

[25] Zhang H, Zhang D, Chen J, Yang Y, Huang Z, Huang D, et al Tomato stress-responsive factor TSRF1 interacts with ethylene responsive element GCC box and regulates pathogen resistance to *Ralstonia solanacearum*. Plant Mol Biol. 2004; 55(6): 825–834. doi: 10.1007/s11103-004-2140-8 15604719 10.1007/s11103-004-2140-8

[26] Holzberg S, Brosio P, Gross C, Pogue GP. Barley stripe mosaic virus-induced gene silencing in a monocot plant. Plant J. 2002; 30(3): 315–327. 12000679 10.1046/j.1365-313x.2002.01291.x

[27] Scofield SR, Huang L, Brandt AS, Gill BS. Development of a virus-induced gene-silencing system for hexaploid wheat and its use in functional analysis of the Lr21-mediated leaf rust resistance pathway. Plant Physiol. 2005; 138(4): 2165–2173. doi: 10.1104/pp.105.061861 16024691 10.1104/pp.105.061861 PMC1183404

[28] Livak KJ, Schmittgen TD. Analysis of relative gene expression data using real-time quantitative PCR and the 2(T)(-Delta Delta C) method. Methods. 2001; 25(4): 402–408. doi: 10.1006/meth.2001.1262 11846609 10.1006/meth.2001.1262

[29] Wang CF, Huang LL, Buchenauer H, Han QM, Zhang HC, Kang ZS. Histochemical studies on the accumulation of reactive oxygen species (O_2_– and H_2_O_2_) in the incompatible and compatible interaction of wheat-*Puccinia striiformis* f. sp. *tritici*. Physiol Mol Plant Pathol. 2007; 71(4): 230–239.

[30] Thordal-Christensen H, Zhang ZG, Wei YD, Collinge DB. Subcellular localization of H_2_O_2_ in plants. H_2_O_2_ accumulation in papillae and hypersensitive response during the barley-powdery mildew interaction. Plant J. 1997; 11(6): 1187–1194.

[31] Doke N. Involvement of superoxide anion generation in the hypersensitive response of potato tuber tissues to infection with an incompatible race of phytophthora infestans and to the hyphal wall components. Physiol Plant Pathol. 1983; 23(3): 345–357.

[32] Abbruscato P, Nepusz T, Mizzi L, Del Corvo M, Morandini P, Fumasoni I, et al OsWRKY22, a monocot WRKY gene, plays a role in the resistance response to blast. Mol Plant Pathol. 2012; 13(8): 828–841. doi: 10.1111/j.1364-3703.2012.00795.x 22443363 10.1111/j.1364-3703.2012.00795.x PMC6638809

[33] Dang FF, Wang YN, Yu L, Eulgem T, Lai Y, Liu ZQ, et al CaWRKY40, a WRKY protein of pepper, plays an important role in the regulation of tolerance to heat stress and resistance to *Ralstonia solanacearum* infection. Plant Cell Environ. 2013; 36(4): 757–774. doi: 10.1111/pce.12011 22994555 10.1111/pce.12011

[34] Wang Y, Dang F, Liu Z, Wang X, Eulgem T, Lai Y, et al CaWRKY58, encoding a group I WRKY transcription factor of *Capsicum annuum*, negatively regulates resistance to *Ralstonia solanacearum* infection. Mol Plant Pathol. 2013; 14(2): 131–144. doi: 10.1111/j.1364-3703.2012.00836.x 23057972 10.1111/j.1364-3703.2012.00836.x PMC6638745

[35] Chen L, Zhang L, Li D, Wang F, Yu D. WRKY8 transcription factor functions in the TMV-cg defense response by mediating both abscisic acid and ethylene signaling in Arabidopsis. Proc Natl Acad Sci U S A. 2013; 110(21): E1963–1971. doi: 10.1073/pnas.1221347110 23650359 10.1073/pnas.1221347110 PMC3666684

[36] Lai ZB, Vinod K, Zheng ZY, Fan BF, Chen ZX. Roles of Arabidopsis WRKY3 and WRKY4 transcription factors in plant responses to pathogens. BMC Plant Biol. 2008; 8(1): 1. 18570649 10.1186/1471-2229-8-68 PMC2464603

[37] Chen L, Zhang L, Yu D. Wounding-induced WRKY8 is involved in basal defense in Arabidopsis. Mol Plant Microbe Interact. 2010; 23(5): 558–565. doi: 10.1094/MPMI-23-5-0558 20367464 10.1094/MPMI-23-5-0558

[38] Mukhtar MS, Deslandes L, Auriac MC, Marco Y, Somssich IE. The Arabidopsis transcription factor WRKY27 influences wilt disease symptom development caused by *Ralstonia solanacearum*. Plant J. 2008; 56(6): 935–947. doi: 10.1111/j.1365-313X.2008.03651.x 18702671 10.1111/j.1365-313X.2008.03651.x

[39] Xing DH, Lai ZB, Zheng ZY, Vinod KM, Fan BF, Chen ZX. Stress- and pathogen-induced Arabidopsis WRKY48 is a transcriptional activator that represses plant basal defense. Mol Plant. 2008; 1(3): 459–470. doi: 10.1093/mp/ssn020 19825553 10.1093/mp/ssn020

[40] Shen QH, Saijo Y, Mauch S, Biskup C, Bieri S, Keller B, et al Nuclear activity of MLA immune receptors links isolate-specific and basal disease-resistance responses. Science. 2007; 315(5815): 1098–1103. doi: 10.1126/science.1136372 17185563 10.1126/science.1136372

[41] Oh SK, Baek KH, Park JM, Yi SY, Yu SH, Kamoun S, et al Capsicum annuum WRKY protein CaWRKY1 is a negative regulator of pathogen defense. New Phytol. 2008; 177(4): 977–989. doi: 10.1111/j.1469-8137.2007.02310.x 18179600 10.1111/j.1469-8137.2007.02310.x

[42] AbuQamar S, Chen X, Dhawan R, Bluhm B, Salmeron J, Lam S, et al Expression profiling and mutant analysis reveals complex regulatory networks involved in Arabidopsis response to Botrytis infection. Plant J. 2006; 48(1): 28–44. doi: 10.1111/j.1365-313X.2006.02849.x 16925600 10.1111/j.1365-313X.2006.02849.x

[43] Wang D, Amornsiripanitch N, Dong XN. A genomic approach to identify regulatory nodes in the transcriptional network of systemic acquired resistance in plants. PLoS Path. 2006; 2(11): 1042–1050. 10.1371/journal.ppat.0020123 PMC1635530 17096590

[44] Chen X. Review article: high-temperature adult-plant resistance, key for sustainable control of stripe rust. Am J Plant Sciences. 2013; 4(3): 608–627.

[45] Christianson T. Temperature studies with wheat leaf rust. Can J Plant Pathol. 1993; 15 (2): 97–101.

[46] Plotnikova L Y, Stubei T Y. Effectiveness of the wheat *Lr22b*, *Lr34*, and *Lr37*, genes for adult plant resistance to leaf rust in West Siberia and the cytophysiological basis of their action. Russ J Genet: Appl Res. 2013; 3(1): 47–53.47.

[47] Zhang P, Qi A, Zhou Y, Xia X, He Z, Li Z, et al Quantitative trait loci mapping of adult-plant resistance to leaf rust in a fundulea 900×‘thatcher’ wheat cross. Plant Breeding. 2017; 136: 1–7.

[48] Suenaga K, Singh RP, Huerta-Espino J, William HM. Microsatellite markers for genes lr34/yr18 and other quantitative trait loci for leaf rust and stripe rust resistance in bread wheat. Phytopathology. 2003; 93(7): 881–890. doi: 10.1094/PHYTO.2003.93.7.881 18943170 10.1094/PHYTO.2003.93.7.881

[49] Lillemo M, Asalf B, Singh RP, Huerta-Espino J, Chen XM, He ZH, et al The adult plant rust resistance loci Lr34/Yr18 and Lr46/Yr29 are important determinants of partial resistance to powdery mildew in bread wheat line Saar. Theor Appl Genet. 2008; 116(8): 1155–1166. doi: 10.1007/s00122-008-0743-1 18347772 10.1007/s00122-008-0743-1

[50] Fu D, Uauy C, Distelfeld A, Blechl A, Epstein L, Chen X, et al A kinase-START gene confers temperature-dependent resistance to wheat stripe rust. Science. 2009; 323(5919): 1357–1360. doi: 10.1126/science.1166289 19228999 10.1126/science.1166289 PMC4737487

[51] Park C-J, Ronald PC. Cleavage and nuclear localization of the rice XA21 immune receptor. Nat Commun. 2012; 3: 920 doi: 10.1038/ncomms1932 22735448 10.1038/ncomms1932 PMC3621455

[52] Liu J, Chen X, Liang X, Zhou X, Yang F, Liu J, et al Alternative splicing of rice WRKY62 and WRKY76 transcription factor genes in pathogen defense. Plant Physiol. 2016; doi: 10.1104/pp.15.01921 27208272 10.1104/pp.15.01921 PMC4902586

[53] Journot-Catalino N, Somssich IE, Roby D, Kroj T. The transcription factors WRKY11 and WRKY17 act as negative regulators of basal resistance in Arabidopsis thaliana. Plant Cell. 2006; 18(11): 3289–3302. doi: 10.1105/tpc.106.044149 17114354 10.1105/tpc.106.044149 PMC1693958

[54] Brown JK. Yield penalties of disease resistance in crops. Curr Opin Plant Biol. 2002; 5(4): 339–344. 12179968 10.1016/s1369-5266(02)00270-4

[55] Bakshi M, Oelmuller R. WRKY transcription factors: Jack of many trades in plants. Plant Signal Behav. 2014; 9(2): e27700 doi: 10.4161/psb.27700 24492469 10.4161/psb.27700 PMC4091213

[56] Pandey SP, Somssich IE. The role of WRKY transcription factors in plant immunity. Plant Physiol. 2009; 150(4): 1648–1655. doi: 10.1104/pp.109.138990 19420325 10.1104/pp.109.138990 PMC2719123

[57] Pieterse CMJ, Leon-Reyes A, Van der Ent S, Van Wees SCM. Networking by small-molecule hormones in plant immunity. Nat Chem Biol. 2009; 5(5): 308–316. doi: 10.1038/nchembio.164 19377457 10.1038/nchembio.164

[58] Verhage A, van Wees SCM, Pieterse CMJ. Plant immunity: it's the hormones talking, but what do they say? Plant Physiol. 2010; 154(2): 536–540. doi: 10.1104/pp.110.161570 20921180 10.1104/pp.110.161570 PMC2949039

[59] Rojo E, Solano R, Sanchez-Serrano JJ. Interactions between signaling compounds involved in plant defense. J Plant Growth Regul. 2003; 22(1): 82–98.

[60] Thomma BPHJ, Penninckx IAMA, Broekaert WF, Cammue BPA. The complexity of disease signaling in Arabidopsis. Curr Opin Immunol. 2001; 13(1): 63–68. 11154919 10.1016/s0952-7915(00)00183-7

[61] Ward ER, Uknes SJ, Williams SC, Dincher SS, Wiederhold DL, Alexander DC, et al Coordinate gene activity in response to agents that induce systemic acquired resistance. Plant Cell. 1991b; 3(10): 1085–1094. 12324583 10.1105/tpc.3.10.1085 PMC160074

[62] Sol JF, Linthorst HJM, Cornelissen BJC. Plant pathogenesis-related proteins induced by virus infection. Annu. Rev. Phytopathol. 1990; 28: 113–138.63.

[63] Kotchoni SO, Gachomo EW. The reactive oxygen species network pathways: an essential prerequisite for perception of pathogen attack and the acquired disease resistance in plants. J Biosci (Bangalore). 2006; 31(3): 389–404. 10.1007/BF02704112 17006022

[64] Shetty NP, Jorgensen HJL, Jensen JD, Collinge DB, Shetty HS. Roles of reactive oxygen species in interactions between plants and pathogens. Eur J Plant Pathol. 2008; 121(3): 267–280.

[65] Apostol I, Heinstein PF, Low PS. Rapid stimulation of an oxidative burst during elicitation of cultured plant cells: role in defense and signal transduction. Plant Physiol. 1989; 90(1): 109–116. 16666719 10.1104/pp.90.1.109 PMC1061684

[66] Bolwell PP, Page A, Pislewska M, Wojtaszek P. Pathogenic infection and the oxidative defenses in plant apoplast. Protoplasma. 2001; 217(1–3): 20–32. 11732333 10.1007/BF01289409

[67] Tenhaken R, Levine A, Brisson LF, Dixon RA, Lamb C. Function of the oxidative burst in hypersensitive disease resistance. Proc Natl Acad Sci USA. 1995; 92(10): 4158–4163. 11607542 10.1073/pnas.92.10.4158 PMC41903

[68] Thordal-Christensen H, Zhang ZG, Wei YD, Collinge DB. Subcellular localization of H_2_O_2_ in plants. H_2_O_2_ accumulation in papillae and hypersensitive response during the barley-powdery mildew interaction. Plant J. 1997; 11(6): 1187–1194.

[69] Delledonne M, Xia YJ, Dixon RA, Lamb C. Nitric oxide functions as a signal in plant disease resistance. Nature. 1998; 394(6693): 585–588. doi: 10.1038/29087 9707120 10.1038/29087

[70] Huckelhoven R, Fodor J, Preis C, Kogel KH. Hypersensitive cell death and papilla formation in barley attacked by the powdery mildew fungus are associated with hydrogen peroxide but not with salicylic acid accumulation. Plant Physiol. 1999; 119(4): 1251–1260. 10198083 10.1104/pp.119.4.1251 PMC32009

[71] Joseph LM, Koon TT, Man WS. Antifungal effects of hydrogen peroxide and peroxidase on spore germination and mycelial growth of Pseudocercospora species. Can J Bot. 1998; 76(12): 2119–2124.

[72] Leon J, Awton ML, Raskin I. Hydrogen peroxide stimulates salicyclic acid biosynthesis in tobacco. Plant Physiol. 1995; 108(4): 1673–1678. 12228572 10.1104/pp.108.4.1673 PMC157549

[73] Chen B, Niu F, Liu WZ, Yang B, Zhang J, Ma J, et al Identification, cloning and characterization of R2R3-MYB gene family in canola (*Brassica napus* L.) identify a novel member modulating ROS accumulation and hypersensitive-like cell death. DNA Res. 2016; 23(2): 101–114. doi: 10.1093/dnares/dsv040 26800702 10.1093/dnares/dsv040 PMC4833418

[74] Williams B, Dickman M. Plant programmed cell death: can't live with it; can't live without it. Mol Plant Pathol. 2008; 9(4): 531–544. doi: 10.1111/j.1364-3703.2008.00473.x 18705866 10.1111/j.1364-3703.2008.00473.x PMC6640338

[75] Alvarez ME, Pennell RI, Meijer PJ, Ishikawa A, Dixon RA, Lamb C. Reactive oxygen intermediates mediate a systemic signal network in the establishment of plant immunity. Cell. 1998; 92(6): 773–784. 9529253 10.1016/s0092-8674(00)81405-1

