# Supplementary material for: The wheat WRKY transcription factors TaWRKY49 and TaWRKY62 confer differential high-temperature seedling-plant resistance to Puccinia striiformis f. sp. tritici
Source: PLoS One. 2017 Jul 25;12(7):e0181963. doi: 10.1371/journal.pone.0181963 (PMC5526533; doi:10.1371/journal.pone.0181963)
Supplement: S1 Table — (DOC) [file pone.0181963.s001.doc]

Table S1. Primers used in this study.

| Primer Name | Function | Primer sequences (5’-3’) |
| --- | --- | --- |
| GSP5 | 5’RACE of *TaWRKY49* | TCGTGCTCGGCAGCGGTTCT |
| GSP3 | 3’RACE of *TaWRKY49* | TTCTGCTGCTGCCGTTGATGC |
| TaWRKY49cDNA  TaWRKY62VIGS  TaWRKY62Q  TaWRKY62cDNA  TaWRKY49Q  TaWRKY49VIGS  TaPR1.1Q  TaAOSQ  TaPIE1Q  TaPODQ  TaCATQ  Ta26SQ | Full cDNA of *TaWRKY49*  VIGS vector construction of *TaWRKY62*  RT-qPCR of TaWRKY62  Full cDNA of *TaWRKY62*  RT-qPCR of *TaWRKY49*  VIGS vector construction of *TaWRKY49*  RT-qPCR of *TaPR1.1*  RT-qPCR of *TaAOS*  RT-qPCR of *TaPIE1*  RT-qPCR of *TaPOD*  RT-qPCR of *TaCAT*  RT-qPCR of *Ta26S* | F: CTTGTTCCTGGACGAGGAGCCCA  R: ATTACCTGACCTTCTTCATGGCGG  F: ATATTAATTAAAACAGCCAGTGTAGCAATATCTCAGTG  R: TATGCGGCCGCTTACATCTATCGCCCAAGCCG  F: TCGTTGACCACCACCAG  R: AGCCGTCCCCAAATCCA  F: GAGGTCGTCGCCCAGATGTT  R: GTATTCTTACCAGCCTCCAAACTT  F: CTTCCCTGCCGCATTCT  R: ACGCTCTCGCCCTAGTG  F: ATATTAATTAATCGTCCAGGAACAAGAGA  R: TATGCGGCCGCGTGCTCGGCAGCGGTTC  F: ACTACGACTACGGGTCCAACA  R: TCGTAGTTGCAGGTGATGAAG  F: ACCGTGTTCAACAGCTACGG  R: AGCGCCTCTATCGTCACCTT  F: GGAGCCACCAGTCCGTATGA  R: CACCCGGCAGAGGTATTCAA   | F: TCCGTTGTCGCCTCTGGT | | --- |   R: GTGCCTTGCCGATGGTGT  F: TGCCTGTGTTTTTTATCCGAGA  R: CTGCTGATTAAGGTGTAGGTGTTGA  F: GCTGGCTCGTTCAACTGATG  R: GGACCAAGCGTTCTGATTACTC |
