# Supplementary material for: The wheat WRKY transcription factors TaWRKY49 and TaWRKY62 confer differential high-temperature seedling-plant resistance to Puccinia striiformis f. sp. tritici
Source: PLoS One. 2017 Jul 25;12(7):e0181963. doi: 10.1371/journal.pone.0181963 (PMC5526533; doi:10.1371/journal.pone.0181963)
Supplement: S2 Table — Gh: Gossypium hirsutum; Tu: Triticum urartu; Hv: Hordeum vulgare; Hvpp: Hordeum vulgare predicted protein; Os: Oryza sativa Indica Group; SbSORBIDRAFT_03g039550: Sorghum bicolor hypothetical protein SORBIDRAFT_03g039550; PtPOPTR_0006s08730g: Populus trichocarpa hypothetical protein POPTR_0006s08730g; Tc: Theobroma cacao; Ta: Triticum aestivum; At: Arabidopsis thaliana; Aet: Aegilops tauschii; Mt: Medicago truncatula; Me: Manihot esculenta; OSJ: Oryza sativa Japonica Group. Gh: Gossypium hirsutum; Tu: Triticum urartu; Hv: Hordeum vulgare; Hvpp: Hordeum vulgare predicted protein; Os: Oryza sativa Indica Group; SbSORBIDRAFT_03g039550: Sorghum bicolor hypothetical protein SORBIDRAFT_03g039550; PtPOPTR_0006s08730g: Populus trichocarpa hypothetical protein POPTR_0006s08730g; Tc: Theobroma cacao; Ta: Triticum aestivum; At: Arabidopsis thaliana. (DOC) [file pone.0181963.s002.doc]

Table S2. The proteins used for alignment.

| Protein | GenBank or Reference Sequence Number |
| --- | --- |
| GhWRKY21 | AGV75936.1 |
| GhWRKY98 | AIE43875.1 |
| TuWRKY49 | EMS52311.1 |
| Hvpp | BAJ93890.1 |
| HvWRKY36 | ABI13412.1 |
| OsI_04396 | EEC71781.1 |
| TaWRKY49 | LC169122 |
| SbSORBIDRAFT_03g039550 | XP_002458749.1 |
| PtPOPTR_0006s08730g | XP_002309066.2 |
| TcWRKY49 | XP_007026988.1 |
| AtWRKY36 | NP_564976.1 |
| AtWRKY9 | NP_176982.1 |
| AtWRKY6 | NP_564792.1 |
| AtWRKY31 | NP_567644.1 |
| AtWRKY60 | NP_180072.1 |
| AtWRKY18 | NP_567882.1 |
| AtWRKY40 | NP_178199.1 |
| AtWRKY34 | NP_194374.1 |
| AtWRKY33 | NP_181381.2 |
| HvWRKY46 | AAQ63880.1 |
| AtWRKY48 | AED95824.1 |
| AtWRKY8 | AED95372.1 |
| AtWRKY28 | AEE84006.1 |
| AtWRKY49 | AED94939.1 |
| AtWRKY22 | NP_192034.1 |
| AtWRKY29 | NP_194086.3 |
| AtWRKY14 | NP_564359.1 |
| AtWRKY35 | NP_181029.1 |
| AtWRKY46 | NP_182163.1 |
| AtWRKY70 | NP_191199.1 |
| AtWRKY30 | NP_568439.1 |
| AtWRKY41 | NP_192845.1 |
| AtWRKY53 | NP_194112.1 |
| AtWRKY17 | NP_565574.1 |
| AtWRKY7  TaWRKY62  OsWRKY54  OsWRKY89  OsWRKY75  OsWRKY20  AtWRKY62  OsWRKY64 | NP_194155.1  LC169123  BK005057.1  AY781112.1  BK005078.1  BK005023.1  NP_195810.2  DAA05129.1 |

| OsWRKY40 | DAA05105.1 |
| --- | --- |
| AetWRKY55 | EMT18620.1 |
| Os12g0116700 | BAF29015.2 |
| TcWRKY70 | XP_007033512.1 |
| OsWRKY | AAV41384.1 |
| OsJWRKY | ABG21854.1 |
| MtWRKY | XP_003623634.1 |
| MeWRKY18 | AMO00386.1 |
