# Supplementary material for: The wheat WRKY transcription factors TaWRKY49 and TaWRKY62 confer differential high-temperature seedling-plant resistance to Puccinia striiformis f. sp. tritici
Source: PLoS One. 2017 Jul 25;12(7):e0181963. doi: 10.1371/journal.pone.0181963 (PMC5526533; doi:10.1371/journal.pone.0181963)
Supplement: S1 Fig — (A) Agarose gel (2%) electrophoresis showing amplification of a single product of the expected size for all tested genes. M represents the DL2000 DNA marker. (B) Dissociation curves with single peaks generated for all genes. (C) The efficiency of primer-specific polymerase chain reaction (PCR) amplifications. (PDF) [file pone.0181963.s004.pdf]

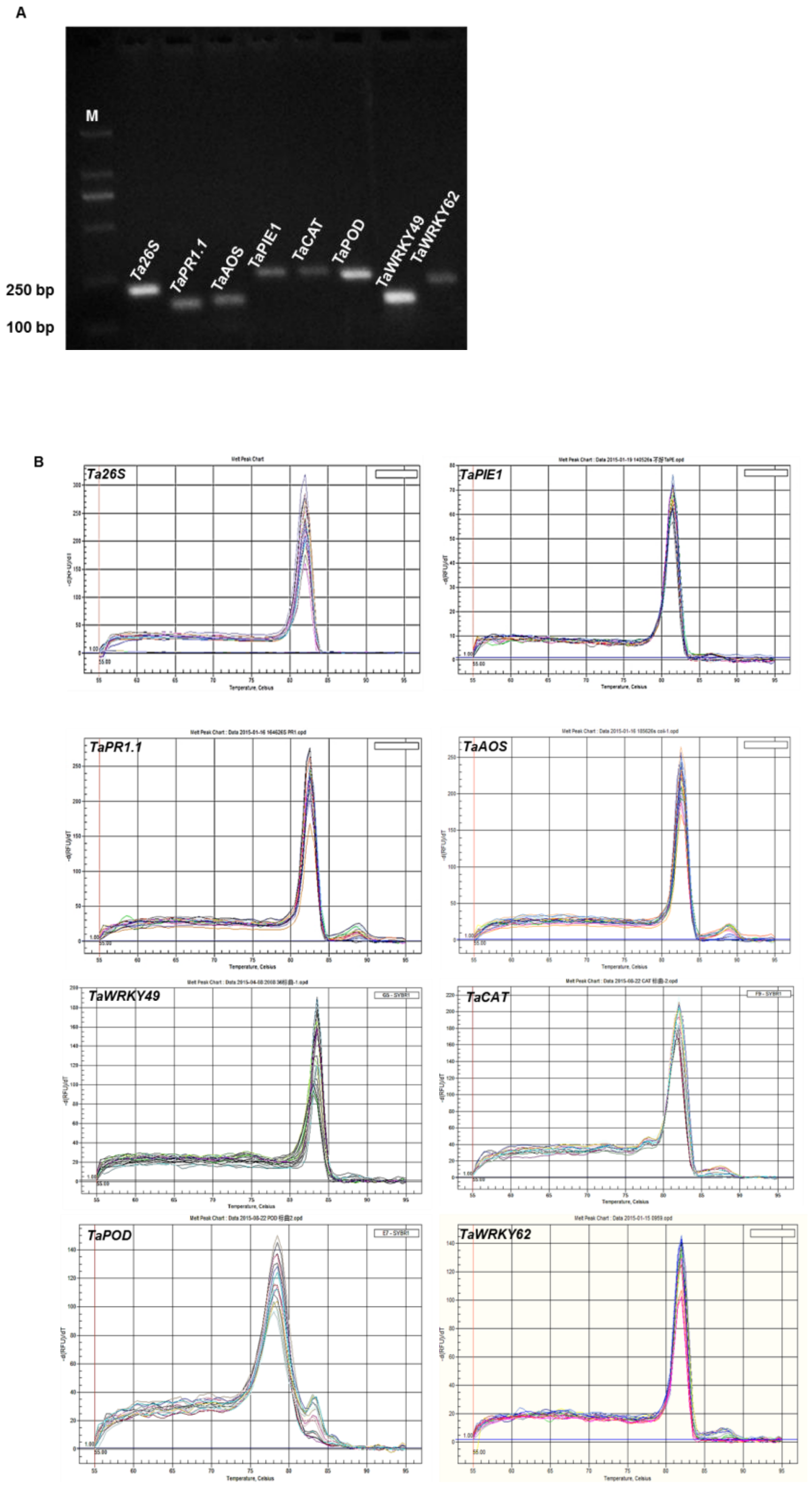
A
M
250 bp
100 bp
Ta26S
TaPR1.1
TaAOS
TaPIE1
TaCAT
TaPOD
TaWRKY49
TaWRKY62
B
Ta26S
TaPIE1
TaPR1.1
TaAOS
TaWRKY49
TaCAT
TaPOD
TaWRKY62

C

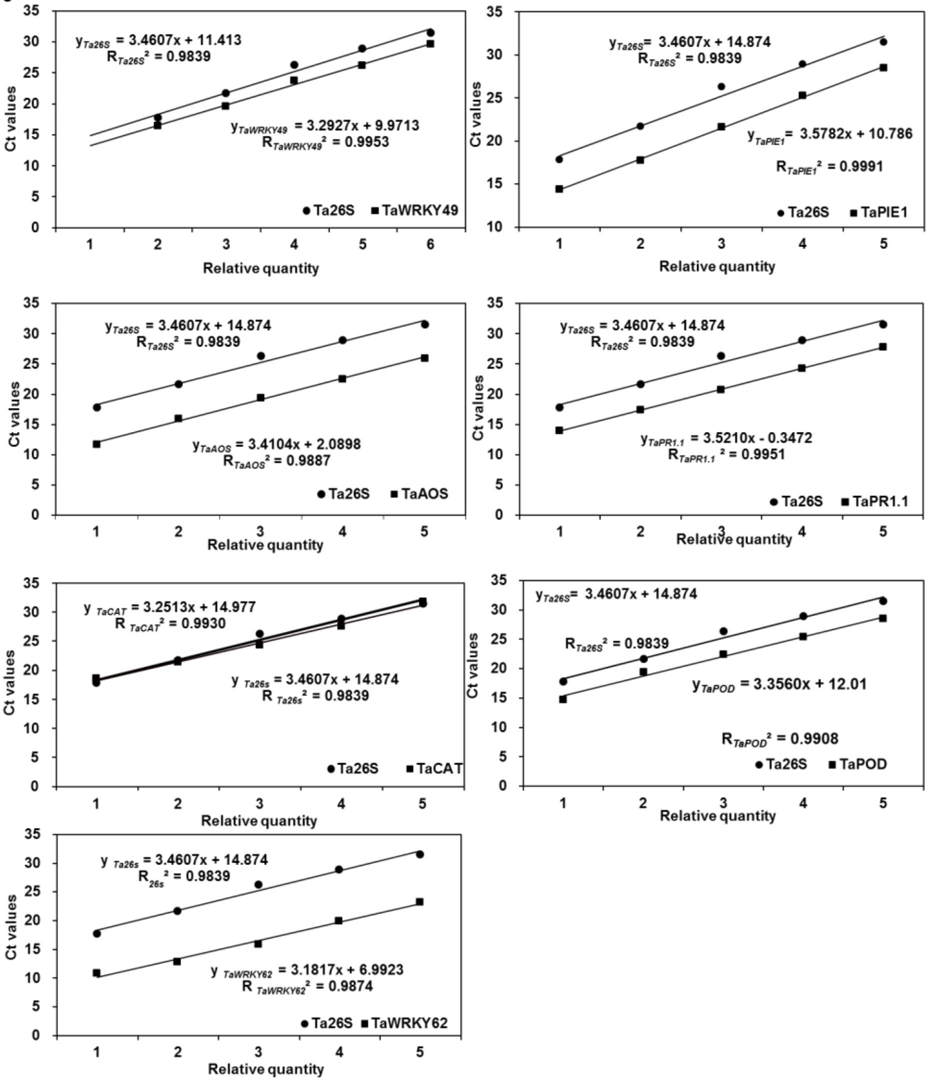

Gene	R^2	Slope	E
TaWRKY49	**0.9953**	**3.2927**	**101.2%**
TaPIE1	**0.9991**	**3.5782**	**90.3%**
TaAOS	**0.9887**	**3.4104**	**96.4%**
TaPR1.1	**0.9951**	**3.5210**	**92.3%**
Ta26S	**0.9839**	**3.4607**	**94.5%**
TaWRKY62	**0.9874**	**3.1817**	**90.3%**
TaCAT	**0.9930**	**3.2513**	**103.0%**
TaPOD	**0.9908**	**3.3560**	**98.6%**

$E=10^{(1/slope)}-1$
