# Supplementary material for: The wheat WRKY transcription factors TaWRKY49 and TaWRKY62 confer differential high-temperature seedling-plant resistance to Puccinia striiformis f. sp. tritici
Source: PLoS One. 2017 Jul 25;12(7):e0181963. doi: 10.1371/journal.pone.0181963 (PMC5526533; doi:10.1371/journal.pone.0181963)
Supplement: S2 Fig — The WRKYGQK peptide stretch is shown in red. The zinc-finger-like motifs in the domains are shown in yellow, the GenBank accession numbers were given in S2 Table. (PDF) [file pone.0181963.s005.pdf]

A

```
Hvpp                       HVTKVRSCGGGKKTPMDGYRWRKYGQKFIKNNPHPRSYYKCTSARCSAKKHVEKSTDDPEMLIVTYEGSHLHGPQ-----210
HvWRKY36                   HVTKVRSCGGGKKTPMDGYRWRKYGQKFIKNNPHPRSYYKCTSARCSAKKHVEKSTDDPEMLIVTYEGSHLHGPQ-----96
TaWRKY49                   HTTKVRGCGGGK-TPMDGYRWRKYGQKFIKNNPHPRSYYKCTSARCSAKKHVEKSTDDPEMLIVTYEGSHLHGPQ-----210
TuWRKY49                   HTTKVRSCGGGK-TPMDGYRWRKYGQKFIKNNPHPRSYYKCTSARCSAKKHVEKSTEDPEMLIVTYEGSHLHGPQ-----200
OsI_04396                  YTSKVRSCGGKM—PADGYKWRKYGQKSIKNNPHPRSYYKCTSSRCSAKKHVEKSTDDPEMLIVTYEGSHHHGPQPLLPP209
SbSORBIDRAFT_03g039550     YTTKVRSCGGKT—PSDGYKWRKYGQKSIKNNPHPRSYYKCTSSRCGAKKHVEKSTEDPEMLMVTYEGPHLHGPQP----222
GhWRKY21                   YTLKVRSCGNGM—ADDGYKWRKYGQKSIKNSPNPRSYYKCTNPRCNAKKQVEKSRDEADTLIITYEGLHLHFVDP----174
GhWRKY98                   YTLKVRSCGNGM—ADDGYKWRIYGQKSIKNSPNPRSYYKCTNPRCNAKKQVEKSRDEADTLIITYEGLHLHFVDP----174
TcWRKY49                   YTLKIKCCGNGM—ADDGYKWRKYGQKSIKNSPNPRSYYKCTNPRCSAKKQVERSRDDPDTLIITYEGLHLHFPYP----273
PtPOPTR_0006s08730g        YTLKLKRCGDGM—ADDGYKWRKYGQKSIKNSPHPRSYYRCTNARCSAKKQVERCSEDPDTLVITYEGLHLHYTYP----175
```

B

```
OsWRKY64 ----DSVSLETPVPHYDGHQWRKYGQKHINNSKHPRSYYRCTYRQEEKCKATKTVQQREDLHDHPVMYTVVYYGQHTCCKGPVAS-ADDHVVVEASQ--233
Os12g0116700 DSVSLETPVPHYDGHQWRKYGQKHINNSKHPRYVIRHS--------------------------------------------------------172
OsWRKY40     -----------HYDGHQWRKYGQKHIKNSKHPRSYYRCTYRQEEKCKATKTVQQREDLHNGDHPIMYTVVYYGQHTCCKGPAAL-ADDHVVVEA- 232
TaWRKY62      DSVALETPVPHYDGHQWRKYGQKIINHTKHPRSYYKCTYKQDQDCRATKTVQQQQQDAVGDDDPAMYTVVYYGQHTCKPG----QTDAAVVETA 163
OsWRKY      --HTGSTVAQAPHNDGHQWRKYGQKWISRAKHSRSYYRCANSKVQGCPATKTVQQDSSG------SKLFNVDYYDQHTCRGDG----IADPYVVD- 183
OsJWRKY       HTGSTVAQAPHNDGHQWRKYGQKWISRAKHSRSYYRCANSKVQGCPATKTVQQMDSSG-----SKLFNVDYYGQHTCRGDG---IANPYVVDTA137
TcWRKY70     ------------DDGHAWRKYGQKVILNAKHPRNYYRCTHKHDQGCQATKQVQQIEDD------PPKYGTTYYGHHTCKNLLKASQLILDS-TSK 204
MeWRKY18     -----------------WRKYGQKVILNAKHPRNYFRCTHKFDQGCQATKQVQRIEDE——————PPMYRTTYYGHHTCKNLVKPCHLFLDASDDA 221
MtWRKY      ------------------WRKYGQKKILHTDFPRNYYRCTHKHDQGCKATKQVQQIQED------PPLYKTTYYAHHTCR-ILQSSEIIMDSPCDQ 210
AetWRKY55   ----RSLVTNVPHYDGHQWRKYGQKNINGRQHARSYYRCTYT-ERNCSATKTVQQQDQDGGSSIYSADAGEDQGAKYTVVYYGDHTCKASDN—IS 270
```
