# Supplementary figures and images for: The wheat WRKY transcription factors TaWRKY49 and TaWRKY62 confer differential high-temperature seedling-plant resistance to Puccinia striiformis f. sp. tritici
Source: PLoS One. 2017 Jul 25;12(7):e0181963. doi: 10.1371/journal.pone.0181963 (PMC5526533; doi:10.1371/journal.pone.0181963)

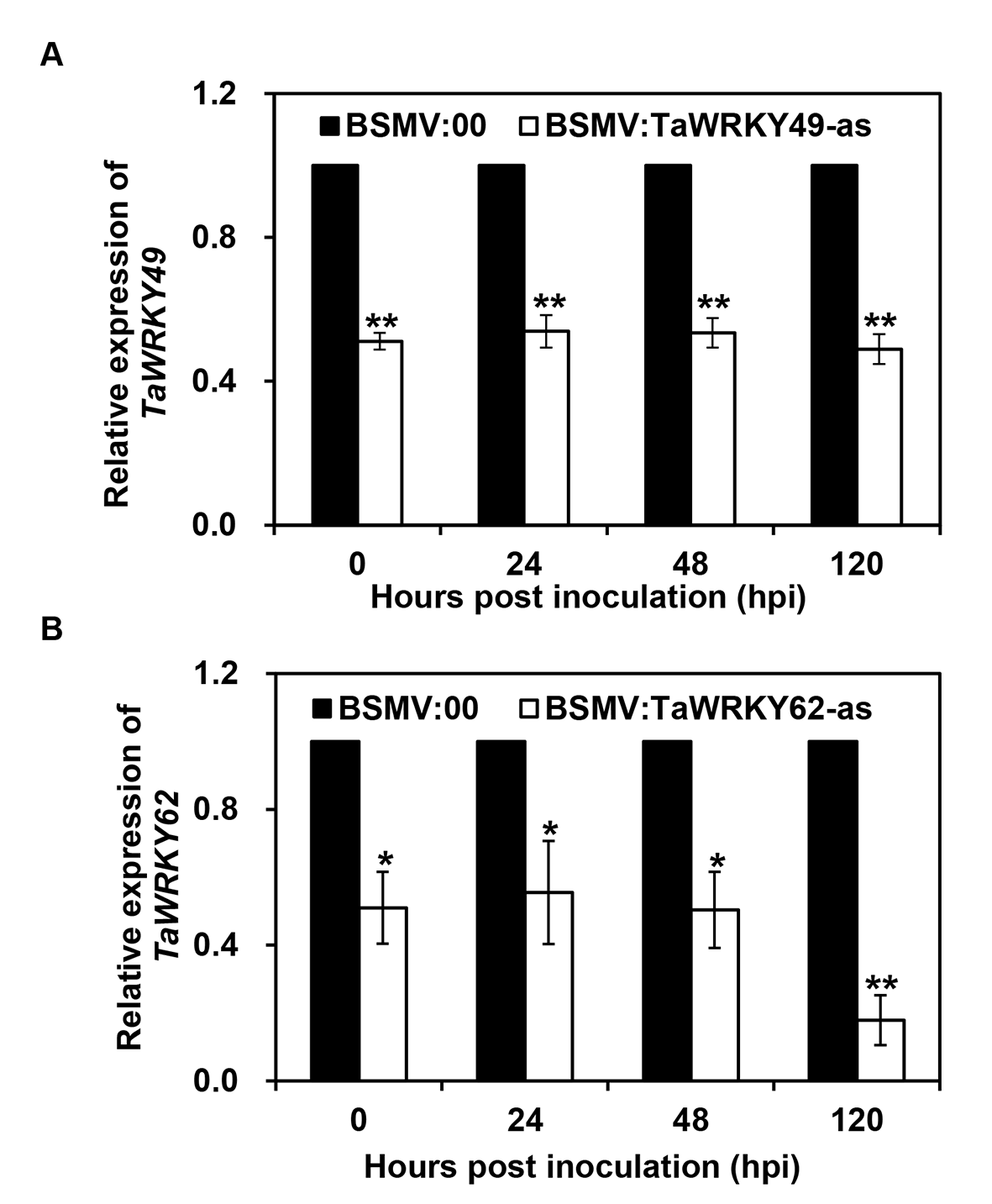

Supplement: S3 Fig — **(Student’s t-test, P < 0.01) or *(Student’s t-test, P < 0.05) indicate significant differences in the mean of gene expression level between the BSMV: WRKY49/62-as-inoculated plants and the BSMV: 00-inoculated plants. Error bars indicate standard error. (TIF) [file pone.0181963.s006.tif]

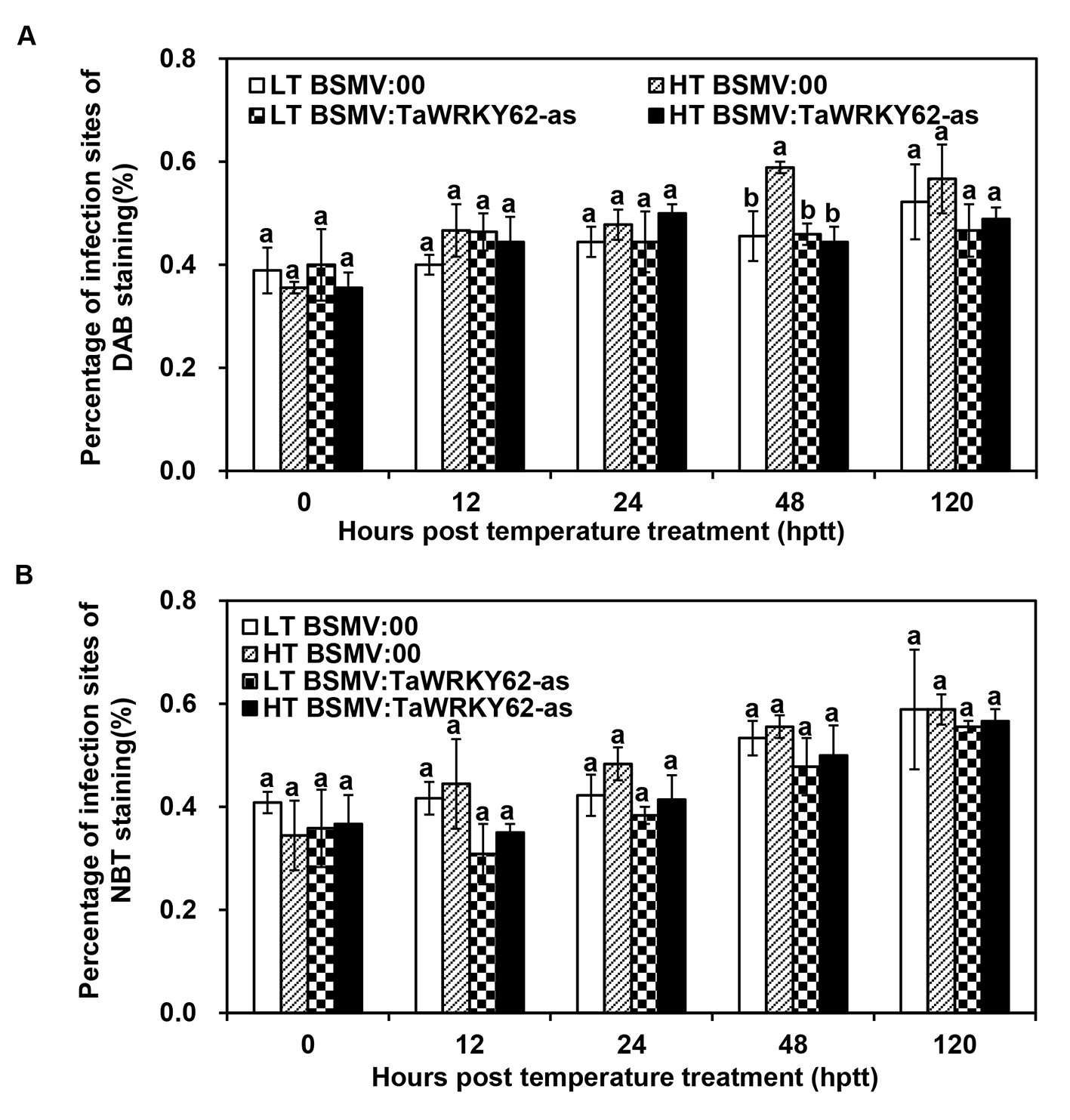

Supplement: S4 Fig — Percentages of infection sites exhibiting (A) H2O2 and (B) O2− accumulation in TaWRKY62-silenced leaves in exposure to HT and LT after inoculation with Pst. 0 hptt: 192 hours post inoculation (hpi) from which HT was applied. Error bars indicate standard error. (TIF) [file pone.0181963.s007.tif]
